# Extracellular vesicle-circEHD2 promotes the progression of renal cell carcinoma by activating cancer-associated fibroblasts

**DOI:** 10.1186/s12943-023-01824-9

**Published:** 2023-07-22

**Authors:** Tao He, Qiansheng Zhang, Peng Xu, Wen Tao, Fuyang Lin, Renfei Liu, Mingzhao Li, Xiaolu Duan, Chao Cai, Di Gu, Guohua Zeng, Yongda Liu

**Affiliations:** 1 Department of Urology, The First Affiliated Hospital of Guangzhou Medical University, 151 West Yanjiang Road, Guangzhou, 510120 China; 2 Urology Key Laboratory of Guangdong Province, Guangzhou, 510120 China; 3 Department of Urology, The Second Affiliated Hospital of Guangzhou Medical University, Guangzhou, 510260 China

**Keywords:** RCC, Extracellular vesicles, circEHD2, Progression, ASO

## Abstract

**Background:**

The encapsulation of circular RNAs (circRNAs) into extracellular vesicles (EVs) enables their involvement in intercellular communication and exerts an influence on the malignant advancement of various tumors. However, the regulatory role of EVs-circRNA in renal cell carcinoma (RCC) remains elusive.

**Methods:**

The in vitro and in vivo functional experiments were implemented to measure the effects of circEHD2 on the phenotype of RCC. The functional role of EVs-circEHD2 on the activation of fibroblasts was assessed by collagen contraction assay, western blotting, and enzyme-linked immunosorbent assay (ELISA). The mechanism was investigated by RNA pull-down assay, RNA immunoprecipitation, chromatin isolation by RNA purification, luciferase assay, and co-immunoprecipitation assay.

**Results:**

We demonstrated that circEHD2 was upregulated in RCC tissues and serum EVs of RCC patients with metastasis. Silencing circEHD2 inhibited tumor growth in vitro and in vivo. Mechanistic studies indicated that FUS RNA -binding protein (FUS) accelerated the cyclization of circEHD2, then circEHD2 interacts with tyrosine 3-monooxygenase/tryptophan 5-monooxygenase activation protein eta (YWHAH), which acts as a bridge to recruit circEHD2 and Yes1-associated transcriptional regulator (YAP) to the promoter of SRY-box transcription factor 9 (SOX9); this results in the sustained activation of SOX9. Heterogeneous nuclear ribonucleoprotein A2/B1 (hnRNPA2B1) regulates the package of circEHD2 into EVs, then EVs-circEHD2 transmits to fibroblasts, converting fibroblasts to cancer-associated fibroblasts (CAFs). Activated CAFs promote the metastasis of RCC by secreting pro-inflammatory cytokines such as IL-6. Furthermore, antisense oligonucleotides (ASOs) targeting circEHD2 exhibited a strong inhibition of tumor growth in vivo.

**Conclusions:**

The circEHD2/YWHAH/YAP/SOX9 signaling pathway accelerates the growth of RCC. EVs-circEHD2 facilitates the metastasis of RCC by converting fibroblasts to CAFs. Our results suggest that EVs-circEHD2 may be a useful biomarker and therapeutic target for RCC.

**Supplementary Information:**

The online version contains supplementary material available at 10.1186/s12943-023-01824-9.

## Introduction

Renal cell carcinoma (RCC) is one of the most diagnosed urological malignancies worldwide, accounting for more than 4% of newly diagnosed cancer cases in the United States [1]. Clear cell renal cell carcinoma (ccRCC), the most common type of kidney cancer, comprises approximately 75% of RCC according to its histological classification [2]. Previous studies have reported that approximately 70% of RCC cases are locally or locally advanced at the initial diagnosis [3]. Although surgery is a curative choice in early-stage ccRCC, approximately 30–50% of cases will eventually develop into fatal metachronous distant metastasis [3, 4]. Therefore, the molecular signatures underlying the development and progression of RCC should be further explored.

Circular RNA (circRNA), which differs from its linear counterpart, possesses a unique covalently closed loop structure that endows it with specific regulatory functions during biological processes [5, 6]. CircRNAs were initially viewed as “splicing errors” or byproducts during RNA transcription. However, more than 16,000 significant circRNAs have been identified in more than 2,000 clinical tumor samples by exome capture sequencing [5]. Additionally, owing to their unique circular structure, circRNAs exhibit characteristics of high conservation and stability, which make circRNAs promising biomarkers [5]. Emerging evidence has suggested that circRNAs are involved in the progression of various diseases, including cancer [6–8]. Until now, circRNAs have been demonstrated to play a vital oncogenic or anti-oncogenic roles in multiple cancers, including prostate cancer [9], breast cancer [10],colorectal cancer [11], and hepatocellular carcinoma [12]. In RCC, circRNA-cRAPGEF5 plays an anti-carcinogenic role by sponging miR-27a-3p [13]. CircMET promotes RCC proliferation by interacting with CDKN2A mRNA and miR-1197 [14]. CircPOLR2A accelerates ccRCC cell invasion and angiogenesis by binding to UBE3C and PEBP1 proteins, then the UBE3C/circPOLR2A/PEBP1 protein -RNA ternary complex regulates the ERK signaling pathway, resulting in the progression of ccRCC [15].

Cancer-associated fibroblasts (CAFs), as the predominant cell type in the tumor stroma, play a crucial role in facilitating the advancement and metastasis of cancer [16]. They originate predominantly from tissue-resident fibroblasts and exhibit high heterogeneity, expressing specific markers for identification, such as α-smooth muscle actin (α-SMA) [17]. Furthermore, CAFs are known to play a role in regulating the inflammatory microenvironment by expressing pro-inflammatory genes such as IL-6, IL-8, TGF-β, and Collagen [16–18]. Although there have been extensive studies on the crosstalk between tumor cells and CAFs, however, the underlying mechanisms by which RCC cells activate CAFs to promote metastasis remain unclear.

Extracellular vesicles (EVs) were extracellular particles released by various cell types which contained various bioactive molecules such as lipids, proteins, and nucleic acids (including circRNAs) [19]. Recent studies confirmed that EVs derived from cancer cells could act as messengers by transmitting bioactive molecules to distant organs, thereby inducing a parenchymal signaling response and remodeling the microenvironment of the metastasis site [19–21]. For example, EVs derived from non-small cell lung cancer can deliver LINC00482 to microglial cells, thus promoting microglial M2 polarization and facilitating brain metastasis [22]. In addition, Tgf-β1 in the EVs derived from colorectal cancer could enhance the formation of liver premetastatic immunosuppressive niche and promote early liver metastasis [23]. EVs-hnRNPA1 triggered by SUMOylation in KRASG12D pancreatic ductal adenocarcinoma could facilitate lymphangiogenesis and lymph node metastasis [24]. Nevertheless, the underlying mechanisms of EVs-circRNAs in RCC with metastasis are still elusive.

Here, we identified a novel circRNA termed circEHD2 (circbase ID: hsa_circ_0003146), which was significantly upregulated in ccRCC tissues and correlated with poor prognosis of patients with ccRCC. circEHD2 enhanced the growth of RCC through the circEHD2/YWHAH/YAP/SOX9 pathway. While EVs-circEHD2 promotes the metastasis of RCC by converting fibroblasts to CAFs. Taken together, our results suggest that EVs-circEHD2 plays a crucial role in the progression of RCC, indicating that EVs-circEHD2 may act as a potential therapeutic target for RCC.

## Materials and methods

### Clinical samples

Eighty patients with ccRCC and normal adjacent tissues (NATs) underwent surgical resection at the First Affiliated Hospital of Guangzhou Medical University (Guangzhou, Guangdong, China). The samples were quickly stored in liquid nitrogen at the Biobank of the Urology Key Laboratory of Guangdong Province. All samples were diagnosed by two pathologists independently. None of the patients had received preoperative treatment. Additionally, we collected preoperative serum from all these patients. The use of the specimens was approved by the Ethical Committee of the First Affiliated Hospital of Guangzhou Medical University (Guangzhou, Guangdong, China). Written informed consent was obtained from all of the included patients. The patients’ detailed clinicopathologic characteristics data were summarized in Table S1.

### Cell lines

All human RCC cell lines OSRC-2, 786-O, Caki-1, 769-P, the human renal proximal tubular epithelial cell line (HK2), the mouse RCC cell line Renca, the fibroblast cell line MRC5, and the human embryonic kidney 293T cell line were purchased from the American Type Culture Collection (ATCC, Manassas, VA). All cell lines were cultured at 37 °C with 5% CO2. The OSRC-2, 786-O, 769-P, Renca, HK2, and 293T cell lines were cultured in RPMI 1640 medium (Biosharp, Guangzhou, China), the MRC5 cell line was maintained in MEN medium (Biosharp, Guangzhou, China), and the Caki-1 cell line was maintained in DMEM medium (Bioss, Beijing, China). All media were supplemented with 10% fetal bovine serum (FBS; Gibco, South America).

### Animal study

All of the experiments involving animals were approved by the Animal Ethics Committee of the First Affiliated Hospital of Guangzhou Medical University.

The subcutaneous xenograft model, orthotopic xenograft model, and tail vein of the lung metastasis model were constructed using female BALB/c nude mice. For the subcutaneous tumor model, four-week-old female BALB/c nude mice were randomly divided into the sh-NC group (n = 4) and sh-circEHD2#1 group (n = 4). Approximately 2 × 10^6^ OSRC-2 cells were resuspended in medium containing 40% Matrigel (BD, San Jose, CA, USA), and then injected into the left flank of each mouse. The tumor growth was measured every week, and the tumor volume was calculated according to the formula (length × width^2^/2). All of the mice were euthanized after four weeks, and the tumor weight was recorded. For the orthotopic mouse model, four-week-old female BALB/c nude mice were randomly divided into the sh-NC group (n = 6) and sh-circEHD2#1 group (n = 6). Then, the mice were anesthetized, and ~ 1 × 10^6^ OSRC-2 cells containing 40% Matrigel (BD, San Jose, CA, USA) were injected into the left subrenal capsule. The orthotopic tumor was monitored using the AniView100 in vivo imaging system (BLT, Guangzhou, China) once a week. All of the mice were euthanized after four weeks, and the tumors were collected for further research. For the tail vein metastasis model, approximately 1 × 10^6^ Renca cells stably expressing firefly luciferase were injected into the tail vein of nude mice and randomly divided into three groups (n = 3/group). Then PBS (control), Renca-EVs_Vector_, and Renca-EVs_circEHD2_ were tail vein injected every three days for four consecutive weeks. The metastatic tumor in the lung was measured every week using the AniView100 in vivo imaging system (BLT, Guangzhou, China). All of the mice were euthanized after four weeks, and the lungs were photographed and resected for further research.

For treatment with circEHD2-ASO in vivo, antisense oligonucleotides (ASOs) specifically targeting circEHD2 and ASO-Ctrl were designed by RiboBio (Guangzhou, China). First, approximately 2 × 10^6^ OSRC-2 cells containing 40% Matrigel (BD, San Jose, CA, USA) were subcutaneously injected into the left flank of each mouse and divided into the ASO-Ctrl group (n = 6) and circEHD2-ASO group (n = 6). After the tumor size reached 100 mm^3^, each group was intratumorally injected with ASO-Ctrl (5 nmol) and circEHD2-ASO (5 nmol) every three days for four consecutive weeks. The tumor volume was measured every week, and the mice were euthanized after four weeks. For the orthotopic mouse model, ~ 1 × 10^6^ OSRC-2 cells containing 40% Matrigel (BD, San Jose, CA, USA) were injected into the left subrenal capsule of each mouse. Then, ASO-Ctrl (10 nmol) and circEHD2-ASO (10 nmol) were delivered through the tail vein every three days (n = 4/group). In vivo imaging using the AniView100 system (BLT, Guangzhou, China) was applied to measure the tumor growth once a week. The mice were euthanized after four weeks, and the tumors were collected for further use.

### Isolation EVs from cell medium and serum

To isolate EVs from RCC cell medium, the RCC cells were cultured in a medium supplemented with 10% FBS without EVs. After culturing the RCC cells for 72 h at 37 °C with 5% CO2, the medium was collected and centrifuged at 2,000 g for 10 min, 3,500 g for 20 min, 10,000 g for 1 h, and 120,000 g for 2 h. All centrifugations were carried out at 4 °C. The purified EVs were resuspended in PBS and stored at -80 °C for further use.

To isolate the EVs from serum, approximately 10 ml of blood was collected from ccRCC patients. Then the blood was centrifuged at 3,000 g for 10 min, 15,000 g for 30 min, and 120,000 g for 2 h. All centrifugations were carried out at 4 °C. The purified EVs were resuspended in PBS and stored at -80 °C for further use.

### Plasmid construction and cell transfection

To stably knock down circEHD2, lentiviral vectors carrying two siRNAs specifically targeting circEHD2 were designed and synthesized by GenePharma (Suzhou, China), while the sh-NC vector was used as a control. A lentiviral vector carrying the full length circEHD2 was designed to overexpress circEHD2, which was also synthesized by GenePharma (Suzhou, China), while a control vector with no circEHD2 sequence was used as a control. To select stably transfected cells, OSRC-2 and 786-O cells were retrovirally infected with the lentiviruses combined with 1 µl Polybrene (5 µg/µl) (GenePharma, Suzhou, China). After 72 h, the OSRC-2 and 786-O cells were selected by puromycin at a concentration of 5 µg/ml in medium. After 10 days, the stably transfected cells were constructed when the cells became resistant to puromycin. To obtain stably overexpressing mutated circEHD2 (201–252-nt region), a lentiviral vector carrying full length mutated circEHD2 was designed and synthesized by GenePharma (Suzhou, China). The remaining procedures were conducted as mentioned above. Similar methods were applied to the Renca cells to overexpress circEhd2. si-RNAs targeting YWHAN, SOX9, FUS, and hnRNPA2B1 were designed and synthesized by RiboBio (Guangzhou, China), and SOX9 plasmids and FUS were obtained from RiboBio. The si-RNAs and plasmids were transfected using Lipofectamine 3000 (Invitrogen, Carlsbad, CA, USA) according to the manufacturer’s instructions. qRT–PCR or western blot was used to confirm the knockdown efficiency or overexpression efficiency of circEHD2, YWHAH, SOX9, FUS and hnRNPA2B1 in RCC cells. The sequences of sh-circEHD2s and siRNAs are described in the Table S2.

### Luciferase reporter assay

The mutant (MUT) plasmid and wild-type (WT) plasmid of SOX9 promoter/circEHD2 pre-mRNA were purchased from RiboBio. A dual-luciferase reporter assay kit (Promega, Madison, WI, USA) was used to measure the Firefly and Renilla luciferase activities. The detailed procedure has been mentioned in our previous study [9].

### EVs and circEHD2-ASO internalization

The EVs derived from RCC cells were labeled by using the PKH26 Red Fluorescent Cell Linker Kit (Umibio, Shanghai, China). Then MRC5 cells were incubated with the PKH26-labeled EVs in the dark overnight. After the cells were stained by 4′,6-diamidino-2-phenylindole (DAPI), confocal fluorescence microscopy (Carl Zeiss AG, Jenna, Germany) was used to observe the internalization of EVs.

For the internalization of circEHD2-ASO, Cy3-labeled circEHD2-ASO was incubated with human RCC cells (OSRC-2 and 786-O) in the dark for 24 h. Then, the PKH67 Green Fluorescent cytomembrane Linker Kit (Solarbio, Beijing, China) was used to label the cytomembrane of RCC cells at 4℃ for 20 min. Next, the cells were washed twice with phosphate buffered saline (PBS), before DAPI was used to stain the nuclei of the RCC cells. The internalization of circEHD2-ASO in RCC cells was photographed by confocal fluorescence microscopy (Carl Zeiss AG, Jenna, Germany). The sequence of circEHD2-ASO was described in the Table S2.

### CCK-8 assay, EdU assay, colony formation assay, and transwell assay

The CCK-8 assay, 5-ethynyl2′deoxyuridine (EdU) assay, and colony formation assay were used to measure the proliferation of RCC cells. For CCK-8 assay, 2,500 cells were seeded in a 96-well plate and cultured for 24, 48, and 72 h. After that, each well was supplemented with 10 µL of CCK-8 reagent (Dojindo, Kumamoto, Japan) and incubated for 3 h at 37 °C. The absorbance was measured by using a microplate reader (Bio-Rad, USA). For EdU assay, 5,000 cells were seeded in a 48-well plate containing a climbing slice. After 24 h, each well was supplemented with EdU reagents (Solarbio, Beijing, China) according to the manufacturer’s instructions. Fluorescence microscopy (Olympus, Tokyo, Japan) was used to capture the images. For colony formation assay, 500 cells were plated in a six-well plate and cultured for 3 weeks. Then, 0.1% crystal violet was used to stain the cells and the colonies were counted. The transwell invasion assay and migration assays were performed according to the methods outlined in our previous report [9].

### Collagen contraction assays

First, prepare the collagen mix on ice. 1 ml collagen mix containing 378 ul Type 1 Rat Tail Collagen (Corning, NY, USA), 500 ul 2X DMEM, and 122 ul distilled water. Then, MRC5 cells were resuspended in collagen mix at a concentration of 3 × 10^5^ cells per 100 ul. Subsequently, collagen mix containing MRC5 cells was added to 24-well plates and solidified for 45 min at 37 °C with 5% CO2. The gels were photographed, and the sizes were measured at various time points after incubation with RCC-EVs.

### RNA/gDNA extraction

Total RNA was extracted from RCC and NAT tissues, RCC cell lines and EVs using TRIzol reagent (Invitrogen, Carlsbad, CA, USA) according to the manufacturer’s protocol. In brief, approximately 50 mg tissues or 1 × 10^6^ cells were lysed in 1 ml of TRIzol reagent for 15 min at room temperature. Subsequently, 200 µl chloroform was added to the lysis buffer for 5 min at 4 °C, followed by centrifugation for 15 min at 12,000 g, 4 °C. After centrifugation, the aqueous phase containing the RNA was transferred to a new tube and isopropanol was added to the aqueous phase at an equal volume for 10 min at 4 °C, followed by centrifugation at 12,000 g for 10 min at 4 °C. Next, the pellet containing the RNA was retained, and 1 ml of 75% ethanol was added to the tube, followed by centrifugation at 10,000 g for 10 min at 4 °C. The pellet was retained and mixed with 30–50 µl RNase-free water. Then, the RNA was stored at − 80℃ until further use. The genomic DNA (gDNA) of OSRC-2 and 786-O cells was extracted using the TIANamp Genomic DNA Kit (TIANGEN, Beijing, China) according to the manufacturer’s protocol.

### qRT–PCR

In brief, 500 ng of total RNA was reverse transcribed to cDNA using the PrimeScript RT Reagent Kit (Takara, Tokyo, Japan) on a T100 Thermal Cycler instrument (BIO-RAD, Hercules, California, USA). Next, the cDNA was amplified by the TB Green Premix Ex Taq (Takara) on a Roche LightCycler® 480II PCR instrument (Basel, Switzerland). GAPDH and U6 were used as the internal standard controls. The relative RNA expression was estimated by the 2–ΔΔCT method. The primers used in the study are described in the Table S3.

### Nuclear and cytoplasmic RNA fractionation assays

A Cytoplasmic & Nuclear RNA Purification Kit (NORGEN, Thorold, ON, Canada) was used to separate the nuclear and cytoplasmic RNA fractions. In brief, approximately 1 × 10^6^ OSRC-2 or 786-O cells were trypsinized, before washing twice with PBS. Next, 200-µl ice-cold Lysis Buffer J (NORGEN) was directly added to the cells, followed by gentle tapping and incubating on ice for 5 min. The lysis buffer was centrifuged at 14,000 g for 3 min at room temperature. The cytoplasmic RNA fraction in the supernatant was carefully transferred to a new tube, while the nuclear RNA fraction was retained at the bottom of the tube. Then, 200 µl Buffer SK was added to the cytoplasmic RNA part and 400 µl of Buffer SK was added to the nuclear RNA part. Next, the cytoplasmic RNA fraction and nuclear RNA fraction were added to 200 µl 100% ethanol, followed by vortexing for 10–15 s and centrifuging at 4,000 g for 1 min at room temperature. Subsequently, 400 µl Wash Solution A was separately added to each part, followed by centrifugation at 4,000 g for 1 min at room temperature. Then, 50 µl Elution Buffer E was added to the column and centrifuged at 200 g for 2 min, immediately followed by 14,000 g for 1 min. Finally, the RNA was reverse transcribed to cDNA through the PrimeScript RT Reagent Kit (Takara) using a T100 Thermal Cycler instrument (BIO-RAD, Hercules, California, USA), and the cDNA was stored at − 80 °C for further use.

### Nuclear protein extraction

The Nucleoprotein Extraction Kit (C500009-0050, Sangon Biotech, Shanghai, China) was used to purify the nuclear proteins of OSRC-2 and 786-O cells. Briefly, 5 × 10^6^ RCC cells were collected and washed twice with ice-cold PBS, followed by incubation with Hypotonic Buffer for 10 min at 4 °C. Then the solution was centrifuged at 800 g for 5 min at 4 °C. Subsequently, the supernatant was discarded and 400 µl Hypotonic Buffer was added before centrifuging at 2,500 g for 5 min at 4 °C. Next, the supernatant was discarded, and the pellet was retained, to which a 200-µl lysis buffer was added, followed by centrifuging at 20,000 g for 10 min. Finally, the supernatant containing the nuclear proteins was collected and stored at − 80 °C for further use.

### RNase R treatment

In brief, the RNA was extracted from OSRC-2 and 786-O cells using TRIzol reagent (Invitrogen, Carlsbad, CA, USA) according to the manufacturer’s protocol. Then, RNase R (1.0 U/µg) (Geenseed, Guangzhou, China) was added to 500ng RNA, followed by incubation for 20 min at 37 °C. A further 500 ng of RNA without RNase R was subjected to the same protocol under the same conditions and used as the control. Subsequently, qRT–PCR was used to measure the stability of circEHD2 and the liner mRNA-EHD2.

### Electrophoresis analysis

Electrophoresis was performed in 1% agarose gel. Briefly, 50 ml 1 × TAE buffer containing 0.5-g agarose was heated to boiling, then, after cooling to 70–80 °C, 5 µl of 4 S GelRed (Sangon Biotech, Shanghai, China) was added and fully mixed. The liquid solution was then poured into a mold until the gel was completely cooled. After the agarose gel was solidified, the agarose gel was placed into an electrophoresis tank that contained 1 × TAE buffer. Subsequently, 10 µl of DNA samples mixed with loading buffer were added to each well, and electrophoresis was conducted at 120 V for 30 min. The bands on the gel were photographed by an ultraviolet imaging system.

### Immunohistochemistry (IHC)

For IHC, the paraffin sections were deparaffinized and dehydrated by xylene and a series of graded ethanol according to the procedure described in our previous study [9]. Paraffin-embedded ccRCC tissues, NATs, orthotopic tumor tissues, and mouse metastasis lungs were subject to IHC analysis to determine the protein expression using antibodies against SOX9 (ab185966, 1:1000, Abcam, Burlingame, CA, USA), FUS (ab243880, 1:300, Abcam), Ki67 (ab92742, 1:500, Abcam), and α-SMA (ab32575, 1:100, Abcam) at 4 °C overnight. Subsequently, the tissues were incubated with secondary antibody (GB23303, 1:400, Servicebio, Wuhan, China) for 1 h at room temperature. Finally, the sections were fixed with neutral balata and photographed by a fluorescence microscope (Olympus, Tokyo, Japan). The staining area score was measured as 0, < 5%; 1, 5–25%; 2, 25–50%; 3, 50–75%; and 4, > 75%. The staining intensity score was measured as 0, no staining; 1, weak staining; 2, moderate staining; and 3, intense staining. The total staining score was determined by two independent pathologists, and the total score was calculated by combination of staining intensity and area, where samples with a score ≥ 6 were considered to have high expression, while those with a score < 6 were considered to have a low expression [25].

### In situ hybridization (ISH)

For ISH, a specific probe targeting the splicing site of circEHD2 was labeled with 5′-digoxin (DIG) and 3′- digoxin (DIG), and the probe was designed and synthesized by Servicebio (Wuhan, China). The scramble probe and U6 probe were used as a negative control and internal control, respectively. Briefly, the paraffin sections were deparaffinized and dehydrated by xylene and a series of graded ethanol, followed by incubation with proteinase K at 37 °C for 20 min and with Triton-X100 at 4 °C for 10 min. The sections were incubated with hybridization buffer containing the circEHD2 probe at 37 °C overnight, followed by incubation with the anti-digoxin antibody at 4 °C overnight. Then, the sections were stained with 5-Bromo-4-Chloro-3-Indolylphosphate/Nitroblue Tetrazolium (BCIP/NBT) (Beyotime, Shanghai, China) for 30 min at room temperature, followed by staining with Nuclear fast red (Servicebio, Wuhan, China) for 3 min at room temperature. The images were photographed by an Olympus microscope (Olympus, Tokyo, Japan). The H-score of circEHD2 was assessed as follows: H-score = Σ (P × I), where P represents the percentage of stained cells, and I indicates the staining intensity score as follows: 0 (no staining), 1 (weak staining), 2 (moderate staining), and 3 (intense staining). The probes used for ISH are listed in the Table S4.

### RNA fluorescence in situ hybridization (FISH)

To measure the sub-cellular localization of circEHD2 in RCC cells, and to estimate the expression level and location of circEHD2 in ccRCC tissues and NATs, a Cy3-labeled probe specifically targeting circEHD2 was designed and synthesized by RiboBio (Guangzhou, China). Briefly, OSRC-2 and 786-O cells were trypsinized and resuspended in medium, then approximately 2,000 cells were placed into a 48-well plate with cover glass. When the cells reached 70–90% confluence, PBS was used to wash the cells three times, followed by fixing with 3.7% paraformaldehyde. Next, 0.5% Triton-100 was used to permeate the cells for 10 min at 4 °C. Subsequently, pre-hybridization buffer was added to the well to pre-hybridized cells at 37 °C for 30 min. Then, hybridization buffer containing the circEHD2-FISH probe was added to the cells and incubated at 37 °C overnight in the dark. After hybridization, a series concentration of SSC solution was used to wash the cells, then the nuclei were stained with DAPI for 15 min in the dark. For the ccRCC tissues and paired NATs, the paraffin sections were deparaffinized and dehydrated by xylene and a series of graded ethanol, before treating with proteinase K for 5–10 min at 37 °C. The remaining steps were the same as in RCC cells. Finally, the images were photographed by a confocal fluorescence microscope (Carl Zeiss AG, Jenna, Germany). The circEHD2-FISH probe used in the assay is listed in the Table S4.

### Colocalization of circEHD2 with YWHAH, circEHD2 with hnRNPA2B1, and YWHAH with YAP

Fluorescence staining was used to measure the colocalization of circEHD2 and YWHAH in RCC cells. In brief, approximately 2,000 RCC cells were placed into a 48-well plate with cover glass. When the cells reached 70–90% confluence, the cells were washed three times with PBS, followed by fixing with 3.7% paraformaldehyde. Next, 0.5% Triton-100 was used to permeate the cells for 10 min at 4 °C. Then, the cells were pre-hybridized for 30 min at 37 °C with pre-hybridization buffer, followed by hybridization buffer containing a Cy3-labeled circEHD2-FISH probe (RiboBio) overnight at 37 °C in dark. Subsequently, the cells were permeated with 0.5% Triton-100 for 10 min at 4 °C, followed by incubation with an anti-YWHAH antibody (Abcam) overnight at 4 °C in the dark with mild rotation. After incubation, the cells were washed with PBS and the nuclei were stained with DAPI for 15 min. For the colocalization of circEHD2 with hnRNPA2B1, and YWHAH with YAP in RCC cells, the steps were the same as those outlined for circEHD2 with YWHAH. Finally, the cells were photographed by a confocal fluorescence microscope (Carl Zeiss AG, Jenna, Germany).

### Western blotting

The Total Protein Extraction Kit (KeyGEN, Nanjing, China) was used to extract the total protein in RCC cells, and the concentrations were measured by a BCA protein assay kit (KeyGEN, Nanjing, China) using a previously outlined procedure [9]. Antibodies against SOX9 (ab185966, 1:5000), FUS (ab243880, 1:1000), YWHAH (ab206292, 1:1000), YAP (ab52771, 1:5000), CD9 (ab236630, 1:1000), CD63 (ab59479, 1:1000), CD81 (ab109201, 1:1000), TSG101 (ab125011, 1:1000), α-SMA (ab32575, 1:1000), hnRNPA2B1 (ab31645,1:500), β-Tubulin (ab179513, 1:1000), GAPDH (ab181602, 1:5000), and H3 (ab32356, 1:2000) were obtained from Abcam, and the secondary antibody (GB23303, 1:3000) was provided by Servicebio (Wuhan, China). Chemiluminescent signals were detected using Western ECL Substrate (Advansta, Menlo Park, CA, USA) and images were captured with a ChemiDoc Imaging System (Bio-Rad, Hercules, CA, USA).

### ELISA analysis

The expression level of IL-6 protein secreted in the medium of MRC5 cells incubated with RCC-EVs was estimated using a Human IL-6 ELISA kit (ab178013, Abcam) following the manufacturer’s instructions.

### Serial deletion of the circEHD2 sequence

For serial deletion of the circEHD2 sequence, various deletion sequences of circEHD2 were designed and synthesized by chemical gene synthesis. Then, the plasmids were transfected by Lipofectamine 3000 (Invitrogen, Carlsbad, CA, USA) according to the manufacturer’s instructions.

### RNA pull-down assay

To validate the interaction between circEHD2 and YWHAH, circEHD2 and hnRNPA2B1, a biotin-coupled circEHD2 probe and control probe were designed and synthesized by GenePharma (Suzhou, China). In brief, approximately 1 × 10^7^ OSRC-2 and 786-O cells were lysed and sonicated in a 4 °C water bath for 30 min. Then, 20 µl of the lysate solution was removed to a new tube for RNA input, and 80 µl of the lysate solution was collected for protein input. Subsequently, the probes were added to the lysate and rotated for 16–24 h at room temperature. Then, 100-µl streptavidin magnetic beads (MCE, Monmouth Junction, NJ, USA) were added to the lysis solution and rotated at room temperature for 2–4 h. Subsequently, a magnetic stand was used to collect the magnetic beads, which were then washed five times with washing buffer (containing PMSF, Protease inhibitor, and RNase inhibitor). Then, the magnetic beads were resuspended in 1 ml washing buffer (containing PMSF, Protease inhibitor, and RNase inhibitor), of which 100 µl was collected for RNA purification and 900 µl was collected for protein purification. For RNA extraction, the 100-µl sample was mixed with 5-µl proteinase K (Sangon Biotech, Shanghai, China) and RNA PK buffer, followed by mild rotating at 50 °C for 45 min, then 95 °C for 10 min to break the formaldehyde cross-links. Next, the RNA was purified by TRIzol reagent (Invitrogen, Carlsbad, CA, USA), and the RNA was reverse transcribed to cDNA and stored at − 80℃ for further use. For protein extraction, 300 µl 4× loading buffer was added to the remaining 900-µl sample and incubated at 100 °C for 10 min. Next, the supernatant containing the protein product was separated by the magnetic stand, and the protein was further used for mass spectrometry (MS) analysis and western blot. The sequences of the circEHD2 probe and control probe are listed in the Table S4.

### Silver staining

In brief, the proteins precipitated by the circEHD2 RNA pull-down assay were electrophoretically separated by 10% SDS-PAGE gel, before staining the gel using a Silver stain kit (BL620A, Biosharp, Beijing, China) according to the manufacturer’s instructions.

### RNA immunoprecipitation (RIP) assay

The RIP assay was performed using a RIP kit (Millipore, MA, USA) to verify the interaction between YWHAH and circEHD2, FUS with circEHD2 pre-mRNA, and hnRNPA2B1 with circEHD2. Briefly, approximately 2 × 10^7^ OSRC-2 and 786-O cells were collected and lysed by RIP lysis buffer. The interaction RNA was precipitated by anti-YWHAH antibody (ab206292, Abcam), anti-FUS antibody (ab243880, Abcam), and anti-hnRNPA2B1 antibody (ab31645, Abcam). The anti-IgG antibody (ab172730, Abcam) was used as a negative control. Then, the coprecipitated RNAs were purified by TRIzol reagent (Invitrogen, Carlsbad, CA, USA) and analyzed by qRT–PCR. For the analysis of circEHD2 pre-mRNA, miR-200 was used as a positive control [26].

### Co-immunoprecipitation (co-IP) assay

In brief, approximately 2 × 10^6^ OSRC-2 and 786-O cells were collected and lysed by the RIPA Lysis Buffer (P0013C, Beyotime, Shanghai, China) at 4 °C for 30 min. Then, 5 µg YWHAH antibody (ab206292, Abcam), YAP antibody (ab52771, Abcam), and IgG antibody (ab172730, Abcam) were incubated with the lysis solution at 4 °C overnight, followed by incubation with A/G protein magnetic beads at 4 °C for 2 h. Western blotting was performed to detect the precipitated proteins.

### Chromatin immunoprecipitation (ChIP) assay

The ChIP procedure was performed using the EZ-ChIP assay kit (Millipore, MA, USA) to demonstrate the interaction between YAP and YWHAH proteins with the promoter SOX9. In brief, 2 × 10^6^ RCC cells were collected and fixed with 1% formaldehyde to cross-link proteins to DNA. Then, the cells were washed three times with PBS and lysed with the lysis buffer. Then, the lysis solution (containing PMSF and protease inhibitor) was sonicated for 20–30 min until the DNA was sheared into small fragments. Subsequently, 10% volume of the lysis solution was transferred to a new tube as input, while the remaining lysis solution was incubated with anti-YAP antibody (ab52771, Abcam), anti-YWHAH antibody (ab206292, Abcam), and anti-IgG antibody (ab172730, Abcam) at 4 °C for 16 h, followed by incubation with A/G protein magnetic beads overnight at 4 °C. The retrieved DNA was then measured by qRT–PCR. The ChIP primers are listed in the Table S3.

### Chromatin isolation by RNA purification (ChIRP) assay

The circEHD2 probe was designed to target the back-spliced site of circEHD2 by GenePharma (Suzhou, China), and the control probe was used as a negative control. In brief, 1 × 10^7^ OSRC-2 and 786-O cells were harvested and fixed with 1% formaldehyde, before lysing and sonicating in a 4 °C water bath for 30 min. Then, 10% volume of the lysate was transferred to a new tube as the input. Subsequently, the remaining lysate was mixed with the circEHD2 probe and control probe and rotated for 16–24 h at room temperature. Subsequently, the streptavidin magnetic beads (MCE, Monmouth Junction, NJ, USA) were co-incubated with the lysis solution at room temperature for 2–4 h. Finally, the DNA was purified using the TIANamp Genomic DNA Kit (TIANGEN, Beijing, China) according to the manufacturer’s protocol, and the enrichment of DNA retrieval was estimated by qRT–PCR. The ChIRP probes are listed in the Table S4.

### Data Availability

The sequencing data of circEHD2 are available in the NCBI’s Gene Expression Omnibus (GSE100186 and GSE108735). The downstream gene sequencing data generated by silencing circEHD2 are available in the Supplementary Data files.

### Statistical analysis

All statistical analyses were performed by SPSS 20.0 (SPSS, Chicago, IL, USA), GraphPad Prism 8.0 (GraphPad Software Inc, CA, USA), and R software (version 3.6.1). All of the in vitro experiments were conducted in triplicate, and the results are indicated as the mean ± SD. Student’s t-test, the Mann–Whitney *U* test, one-way analysis of variance, or the chi-squared test was used to analyze the differences between groups. The Kaplan–Meier method and log-rank test were used to evaluate the OS and PFS. Univariate and multivariate Cox regression analyses were performed to estimate the survival data. Pearson correlation analysis was used to assess the correlations between the groups. P-values < 0.05 were considered statistically significant.

## Results

### Identification of circRNAs in ccRCC

**Fig. 1:**
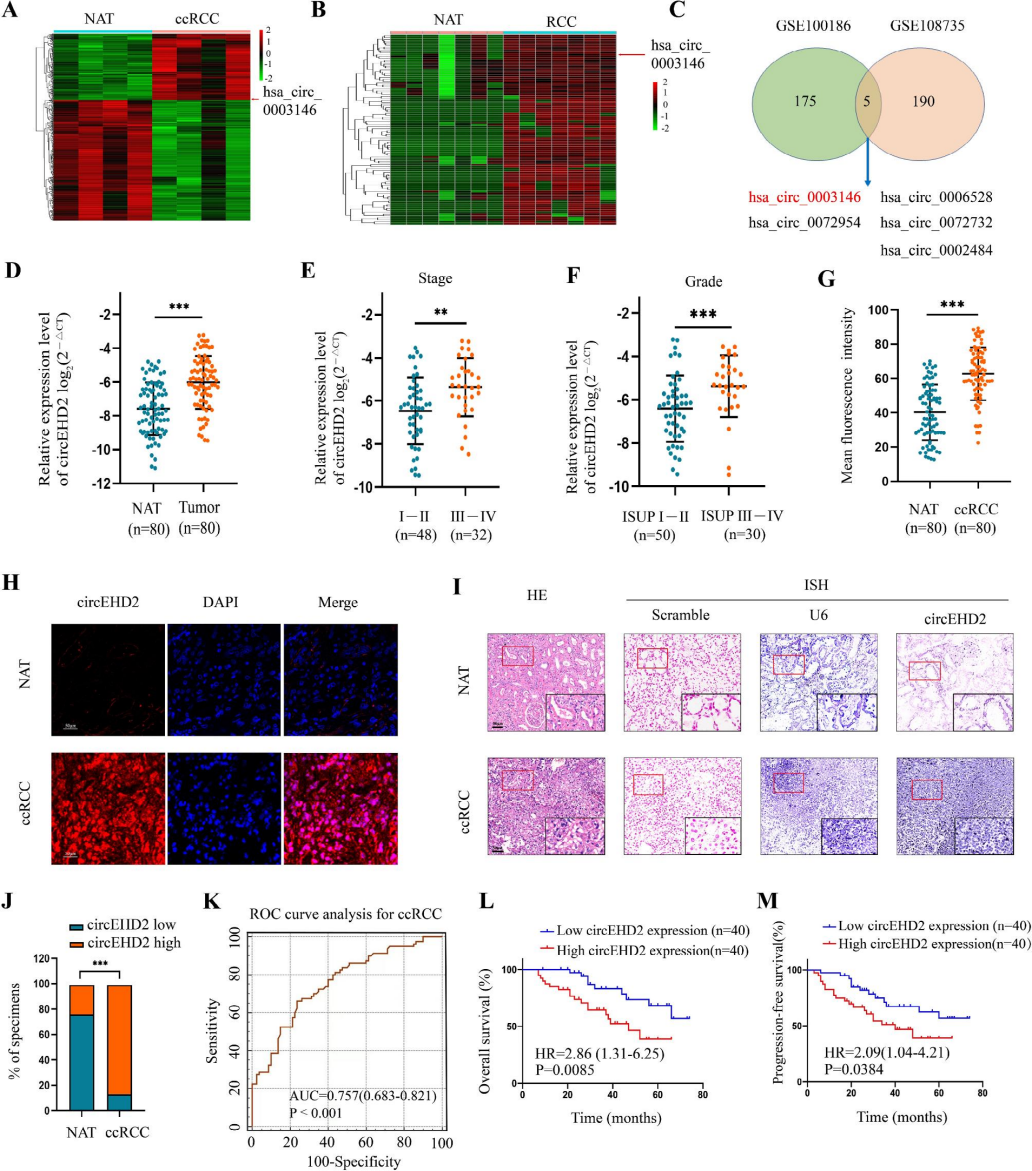
circEHD2 was upregulated in ccRCC tissues and positively associated with poor prognosis. **A** and **B**, The cluster heat maps showed the differentially expressed circRNAs in four ccRCC tissues and paired NATs from GSE100186; and the differentially expressed circRNAs in seven RCC tissues and paired NATs from GSE108735. The red and green strips indicate up-regulated and down-regulated circRNAs, respectively. **C**, The overlapping analysis of upregulated circRNAs in GSE100186 and GSE108735. **D**, The expression level of circEHD2 in 80 ccRCC tissues and paired normal adjacent tissues (NATs) by qRT-PCR. **E** and **F**, The expression level of circEHD2 in a cohort of 80 ccRCC patients stratified by stage and ISUP grade. **G** and **H**, Representative images and quantitative analyses for the circEHD2 expression in ccRCC tissues and paired NATs via FISH. Scale bars: 50 μm. **I**, Representative HE images and ISH images from scramble probe (red: negative control), U6 probe (blue: positive control) and circEHD2 probe in ccRCC tissues and paired NATs. Scale bar: 50 μm. **J**. The percentages of circEHD2 expression in ccRCC tissues and NATs. **K**, ROC curve analysis verified that tissue-circEHD2 could discriminate ccRCC from NAT with high accuracy. **L** and **M**, Kaplan-Meier curves of Overall survival (OS) and Progression-free survival (PFS) according to the level of tissue-circEHD2 in ccRCC patients (n = 80). The cut-off values were the median expression of circEHD2 in ccRCC tissues. **, *P* < 0.01; ***, *P* < 0.001

To investigate the critical circRNAs in ccRCC, a circRNA microarray dataset and a circRNA sequencing dataset of RCC were simultaneously analyzed (GSE100186 and GSE108735). A total of 180 circRNAs were upregulated and 329 circRNAs were downregulated in the tumor tissues compared to the paired normal adjacent tissues (NATs) in GSE100186 (|log2 FC (fold change) ≥ 2| and *P* < 0.05) (Fig. 1A), whereas 195 circRNAs were upregulated in the tumor tissues compared to those in the NATs in GSE108735 (|log2 FC ≥ 2| and *P* < 0.05) (Fig. 1B). Moreover, five circRNAs, including hsa_circ_0003146, hsa_circ_0006528, hsa_circ_0072954, hsa_circ_0072732, and hsa_circ_0002484, were consistently upregulated in both datasets (Fig. 1C and Table S5). Of these five circRNAs, hsa_circ_0003146 had the highest expression level in 30 ccRCC tissues than in those in the paired NATs (Fig. S1A–E); hence, we chose hsa_circ_0003146 for further study.

We termed hsa_circ_0003146 “circEHD2” given that hsa_circ_0003146 is derived from gene-EHD2. circEHD2 is derived from exon-4 of the EHD2 transcript (chr19:48229068–48229481) with a length of 413 bp and is located at the 19q13.13 amplicon (Fig. S2A). Then, rolling circle reverse transcription and Sanger sequencing were conducted to confirm the full-length sequence of circEHD2 in OSRC-2 cells. The results showed that the full-length sequence of circEHD2 was consistent with that in Circbase (Fig. S2B and Fig. S3A–C). To confirm the circular characteristics of circEHD2, convergent and divergent primers were designed to amplify circEHD2 in cDNA and in genomic DNA (gDNA) by reverse transcription PCR (RT-PCR). The gel electrophoresis results showed that circEHD2 could be amplified by both primers in cDNA, whereas circEHD2 could only be amplified by convergent primers in gDNA (Fig. S2C). Additionally, the RNase R digestion assay revealed that circEHD2 was more stable than linear EHD2 mRNA (Fig. S2D-E). The sub-cellular localization analysis of circEHD2 in OSRC-2 and 786-O cells was evaluated via FISH assay and cytoplasmic-nuclear RNA fractionation assay. The results showed that circEHD2 was predominantly enriched in the nucleus (Fig. S2F-G). Taken together, these results demonstrate that circEHD2 possesses a unique circular form and is more stable than its linear counterpart.

### circEHD2 is highly expressed in ccRCC and is positively correlated with ccRCC progression

To explore the clinical relevance of circEHD2 in ccRCC, quantitative real-time PCR (qRT-PCR) was applied to investigate the expression profile of circEHD2 in 80 ccRCC tissues and paired NATs. The results revealed that circEHD2 was dramatically upregulated in ccRCC tissues compared to that in the paired NATs (Fig. 1D). Additionally, we found that a higher circEHD2 level was positively associated with more advanced tumor stage (T3–4) and grade (ISUP III–IV) (Fig. 1E, F). Furthermore, FISH assay showed that circEHD2 was significantly upregulated in ccRCC tissues, whereas circEHD2 was rarely detected in paired NATs (Fig. 1G, H). Similar results were obtained in ccRCC tissues via in situ hybridization (ISH) assays (Fig. 1I, J). Next, we estimated the diagnostic value of tissue-circEHD2 in ccRCC. Receiver operating characteristic (ROC) analysis indicated that circEHD2 could accurately discriminate patients with ccRCC and NAT (Fig. 1K). For survival analysis, Kaplan–Meier survival curve analyses revealed that poor progression-free survival (PFS, P = 0.0384) and poor overall survival (OS, P = 0.0085) were associated with high tissue-circEHD2 levels (Fig. 1L, M). Moreover, univariate and multivariate Cox proportional hazard analyses demonstrated that circEHD2 levels were significantly correlated with OS and PFS, and the level of tissue-circEHD2 was an independent prognostic factor for OS and PFS in patients with ccRCC (Fig. S4A, B). Therefore, our data suggest that circEHD2 may be a novel diagnostic and prognostic factor for patients with ccRCC.

### circEHD2 promotes the proliferation, invasion, and migration of RCC cells

**Fig. 2:**
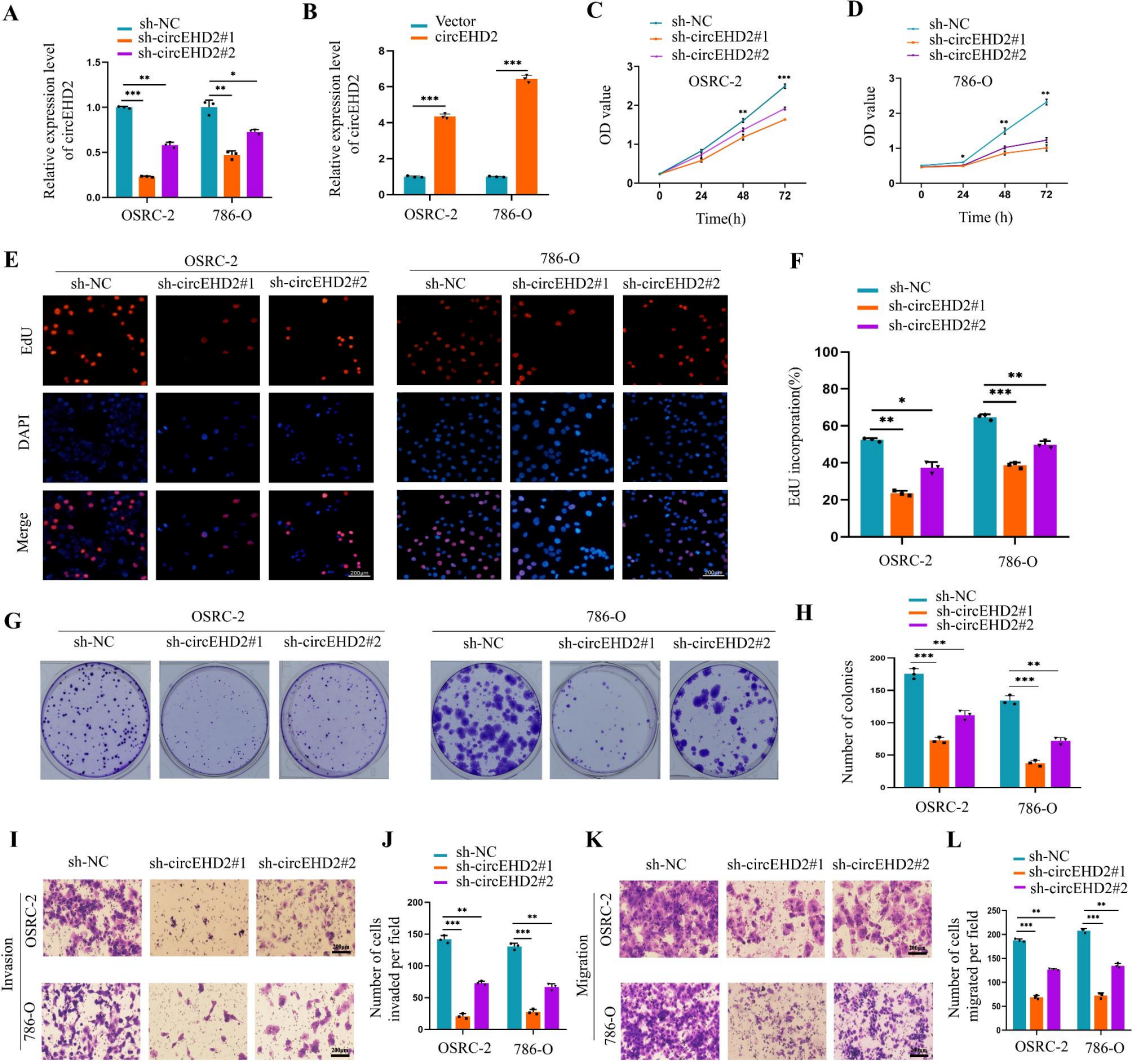
circEHD2 promotes proliferation, migration, and invasion of RCC cells. **A** and **B**, The knockdown efficiency (**A**) and overexpression (**B**) of circEHD2 in RCC cells measured by qRT-PCR. **C** and **D**, The proliferation ability of OSRC-2 (**C**) and 786-O cells (**D**) with circEHD2 knockdown was measured by CCK-8 assays. **E** and **F**, EdU assay revealed that silencing circEHD2 suppressed the proliferation ability of OSRC-2 and 786-O cells. Scale bars: 200 μm. **G** and **H**, The colony formation ability of OSRC-2 and 786-O cells was reduced when transfecting with sh-circEHD2s. **I** and **J**, The invasion abilities of OSRC-2 and 786-O cells were inhibited by the knockdown of circEHD2 through Transwell assays. Scale bars: 200 μm. **K** and **L**, The migration abilities of OSRC-2 and 786-O cells were inhibited by silencing circEHD2 via Transwell assays. Scale bars: 200 μm. Error bars, standard deviation (SD) of three independent experiments. *, *P* < 0.05; **, *P* < 0.01; ***, *P* < 0.001

To explore the expression profile of circEHD2 in RCC cell lines, qRT–PCR was used to measure the expression of circEHD2 across a panel of four RCC cell lines. The results showed that circEHD2 was significantly upregulated in RCC cell lines compared to the human renal proximal tubular epithelial (HK2) cells (Fig. S5A). Therefore, we selected two RCC cell lines (OSRC-2 and 786-O) with relatively high expression levels of circEHD2 for further research. Next, two sh-RNAs that mediated the knockdown of circEHD2 and the full length circEHD2 vector-mediated overexpression of circEHD2 were designed. The results showed that both sh-RNAs stably inhibited the expression of circEHD2, while the overexpression vector obviously increased the expression level of circEHD2 (Fig. 2A, B). However, we found that the mRNA level of EHD2 was not affected by the upregulation or downregulation of circEHD2 (Fig. S5B, C). Subsequently, we found that silencing circEHD2 could significantly inhibit the growth of OSRC-2 and 786-O cells through CCK-8 assays, EdU assays, and colony formation assays (Fig. 2C–H). Additionally, the invasion and migration abilities of OSRC-2 and 786-O cells were significantly inhibited by the knockdown of circEHD2 through transwell assays (Fig. 2I–L). However, the overexpression of circEHD2 in OSRC-2 and 786-O cells enhanced the proliferation capacity through CCK-8 assays, EdU assays, and colony formation assays (Fig. S5D–I). Moreover, the overexpression of circEHD2 in OSRC-2 and 786-O cells could boost the invasion and migration abilities by transwell assays (Fig. S5J–M). Taken together, our results suggest that circEHD2 may exert an oncogenic role in RCC.

### circEHD2 promotes the growth of RCC cells in vivo

**Fig. 3:**
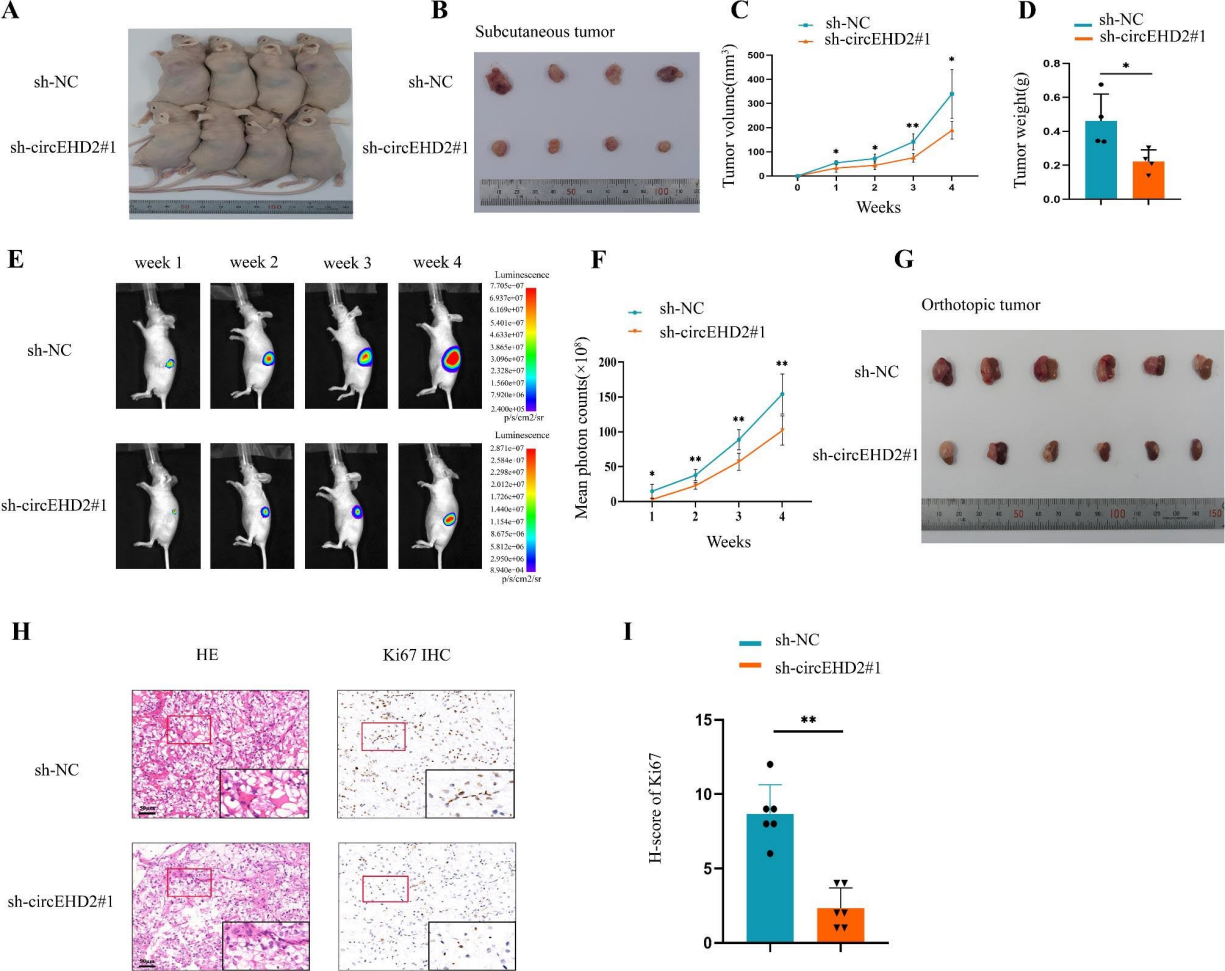
circEHD2 promotes the growth of RCC cells in vivo. **A** and **B**, Images of xenograft tumors after injection of OSRC-2 cells transfected with sh-NC and sh-circEHD2#1 (n = 4/group). **C**, The volumes of subcutaneous tumors were recorded once a week for four consecutive weeks. **D**, The subcutaneous tumor weights were weighed at the endpoint time of the experiment. **E**, The orthotopic tumor growth in the left subrenal capsule was monitored weekly by in vivo bioluminescence imaging(n = 6/group). **F**, The bioluminescence signals of orthotopic tumors were measured by mean photon counts. **G**, Gross appearance of orthotopic tumor after left subrenal capsule injection with sh-NC and sh-circEHD2#1 (n = 6/group). **H**, Images of HE and IHC staining by anti-Ki67 antibody in orthotopic tumors (n = 6/group). Scale bars: 50 μm. **I**, H-score of Ki67 in orthotopic tumors (n = 6/group). *, *P* < 0.05; **, *P* < 0.01

To further validate the oncogenic role of circEHD2 in RCC, the subcutaneous xenograft model and orthotopic xenograft model were established. For the subcutaneous xenograft model, the nude mice were randomly divided into two groups (n = 4/group). Then OSRC-2 cells with stable expression of sh-circEHD2#1 and the control (sh-NC) were injected into the left flank of each mouse. The results showed that the tumor growth was remarkably inhibited in the sh-circEHD2#1 group compared to that in the sh-NC group (Fig. 3A–D). For the orthotopic xenograft model, the nude mice were randomly divided into two groups (n = 6/group). Then, OSRC-2 cells stably expressing sh-circEHD2#1 and the control (sh-NC) were injected into the left subrenal capsule of each mouse. As a result, nude mice injected with sh-circEHD2#1 exhibited weaker bioluminescence signals at each time point (Fig. 3E, F), and the tumor volume was smaller than the sh-NC group (Fig. 3G). Subsequently, the expression level of Ki67 was found to be consistent with circEHD2 in mouse orthotopic tumor tissues by IHC (Fig. 3H, I). Taken together, our data suggest that circEHD2 facilitates the growth of RCC in vivo.

### SOX9 is required for circEHD2-mediated progression in RCC

**Fig. 4:**
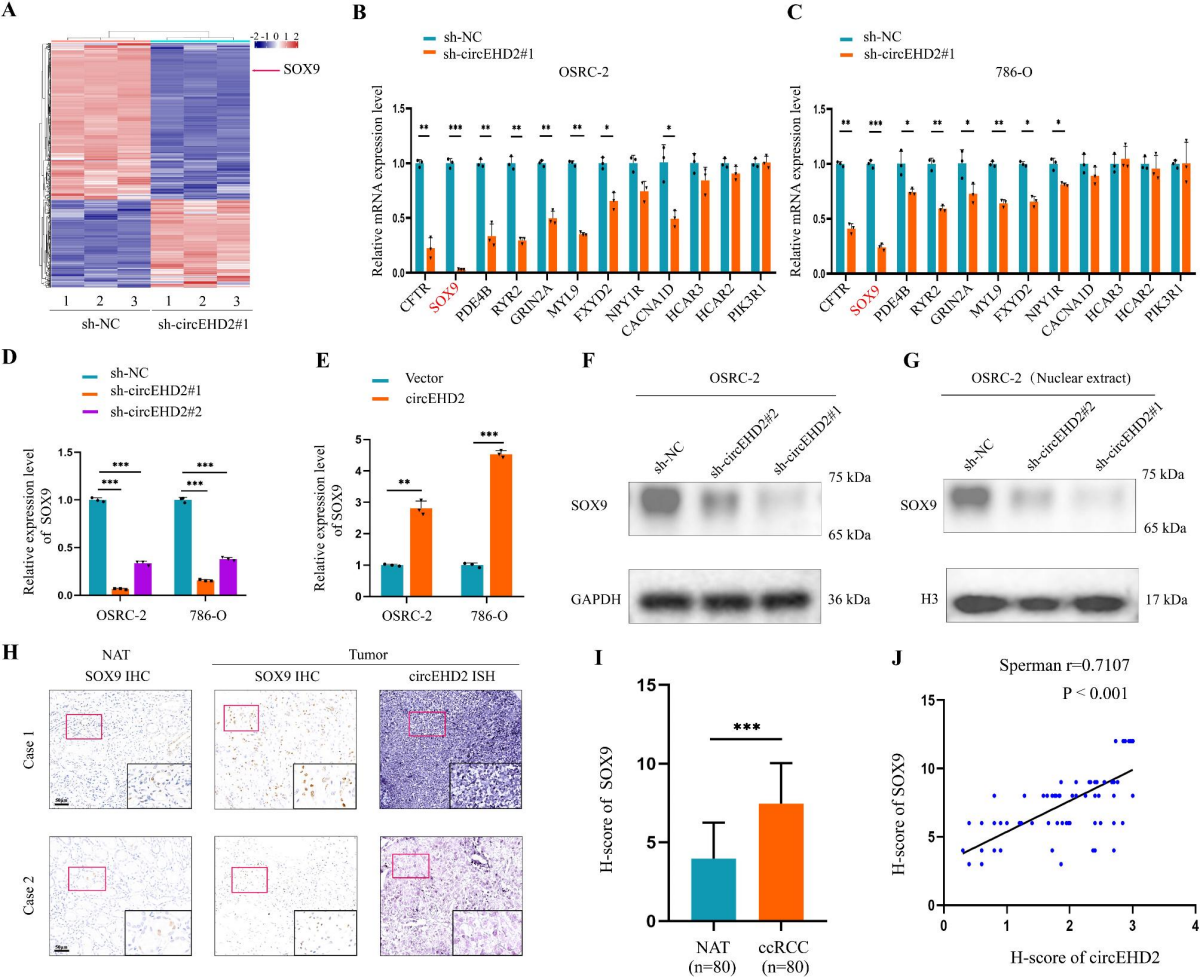
SOX9 is the downstream gene mediated by circEHD2. **A**, The cluster heat maps showed the differentially expressed genes regulated by circEHD2 based on next-generation sequencing (NGS) analysis. The red and blue strips indicate up-regulated and down-regulated genes, respectively. **B** and **C**, qRT–PCR analysis of the cAMP signal pathway-related genes in OSRC-2 (**B**) and 786-O (**C**) cells with circEHD2 knockdown. **D** and **E**, qRT–PCR analysis of the expression level of SOX9 when downregulation (**D**) or upregulation (**E**) of circEHD2 in OSRC-2 and 786-O cells. **F** and **G**, Western blot showed SOX9 expression level in whole protein extracts (**F**) and nuclear protein extracts (**G**) of OSRC-2 cells when silencing circEHD2. **H**, Representative images of IHC and ISH showed the expression of SOX9 and circEHD2 in ccRCC tissues and paired NATs (n = 80). Scale bars: 50 μm. **I**, The H-score of SOX9 in ccRCC tissues and paired NATs (n = 80). **J**, The Pearson correlation analysis showed that circEHD2 expression level was positively correlated with SOX9. Error bars, standard deviation (SD) of three independent experiments. *, *P* < 0.05; **, *P* < 0.01; ***, *P* < 0.001

To explore the downstream genes mediated by circEHD2, Illumina paired-end RNA-seq was performed in the sh-NC group and the sh-circEHD2#1 group. The results revealed that 1,363 dysregulated genes (|log2 fold change ≥ 1| and *P* < 0.05) were regulated by circEHD2, of which 851 genes were downregulated (Fig. 4A and Table S6). Kyoto Encyclopedia of Genes and Genomes (KEGG) pathway enrichment analysis showed that the cAMP signal pathway was one of the most significantly regulated pathways (*P* = 0.0342), with 12 downregulated genes (Fig. S6A). Then, we validated the expression profile of the 12 pivotal genes in the cAMP signaling pathway and found that SOX9 was markedly downregulated in OSRC-2 and 786-O cells when transfected with sh-circEHD2#1 (Fig. 4B, C). Furthermore, qRT–PCR and western blot showed that the expression level of SOX9 was in accordance with overexpression or knockdown of circEHD2 (Fig. 4D–F and Fig. S6B). Similar results were obtained for the nuclear protein extracts (Fig. 4G and Fig. S6C). Moreover, we also found high expression of SOX9 in RCC tissues compared to the paired NATs by IHC (Fig. 4H, I). Consistently, we found a positive correlation between circEHD2 and SOX9 levels (Fig. 4J). The Cancer Genome Atlas (TCGA) database revealed that SOX9 was not only upregulated in kidney renal clear cell carcinoma but also in various types of human cancer (Fig. S6D, E). Collectively, our results provide convincing evidence that SOX9 plays a vital role in the circEHD2-induced progression of RCC.

### circEHD2 directly binds to YWHAH

**Fig. 5:**
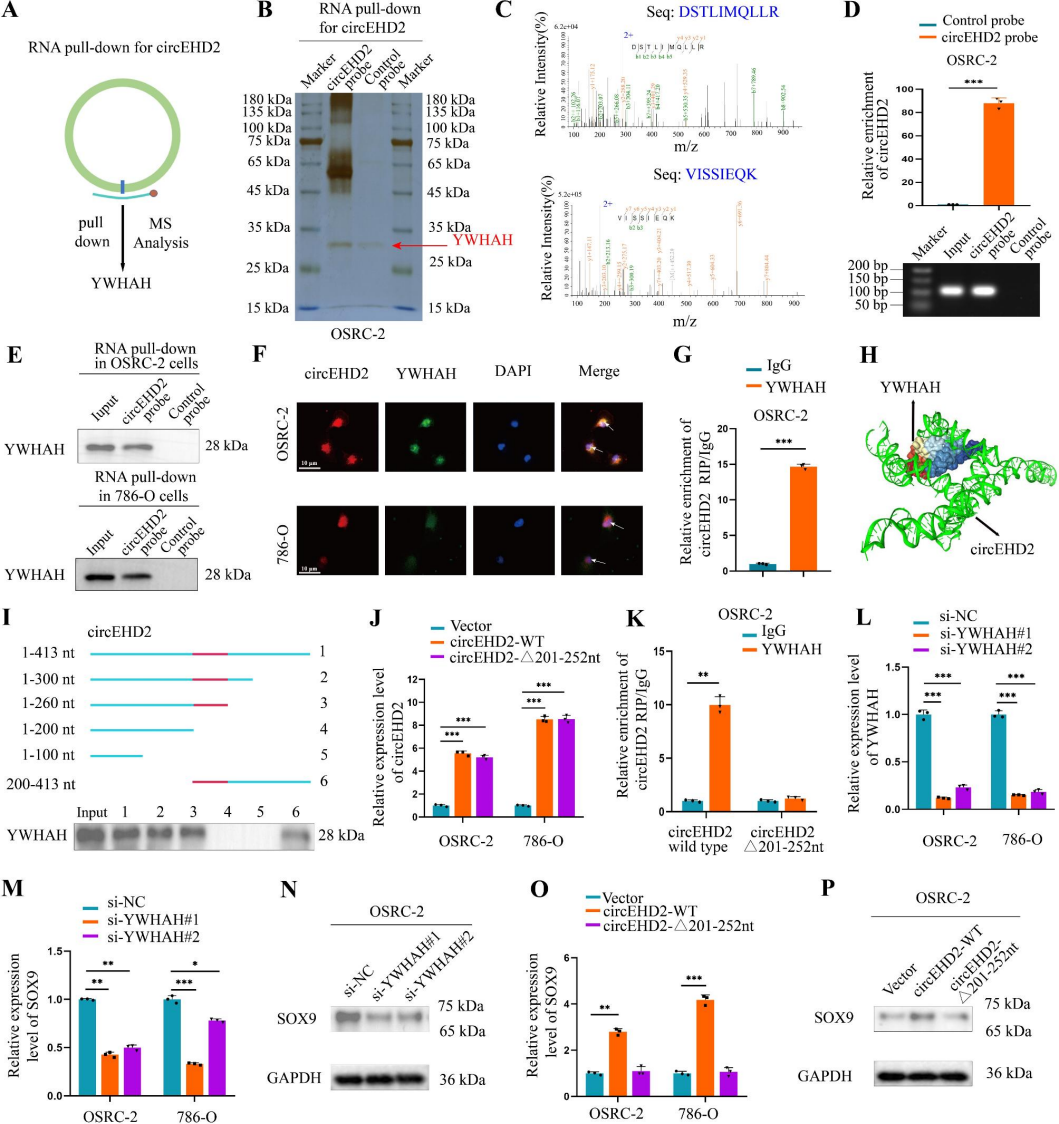
circEHD2 direct interacts with YWHAH. **A**, RNA pull-down assay was applied in RCC cells to identify the proteins that interacted with circEHD2. **B**, The silver staining image of RNA pull-down with circEHD2 probe in OSRC-2 cells. **C**, Mass spectrometry (MS) analysis of the proteins interacted with circEHD2 via RNA pull-down assay. **D**, qRT–PCR analysis confirmed that the circEHD2 probe could specifically enrich circEHD2 in OSRC-2 cells by RNA pull-down assay. **E**, Western blot assay in circEHD2 pull-down proteins confirmed the interaction between circEHD2 and YWHAN. **F**, The subcellular co-localization of circEHD2 and YWHAH in RCC cells was measured by fluorescence staining assay. Scale bars: 10 μm. **G**, RIP assay in OSRC-2 cells confirmed that circEHD2 could be enriched by YWHAH. IgG was used as a negative control. **H**, The three-dimensional structure showed circEHD2 could be bound to YWHAH. **I**, Serial deletions of circEHD2 were used in RNA pull-down assays to confirm the regions of circEHD2 that were required for YWHAH. **J**, qRT–PCR analysis of the level of circEHD2 when mutated the binding sites of circEHD2. **K**, RIP assay was performed in OSRC-2 cells after mutating the 201-252-nt region of circEHD2. **L**, The knockdown efficiency of YWHAH in RCC cells measured by qRT-PCR. **M**, qRT–PCR analysis of the level of SOX9 when knockdown of YWHAH in RCC cells. **N**, Western blot analysis of the expression of SOX9 when downregulation of YWHAH in OSRC-2 cells. **O**, qRT–PCR analysis of the level of SOX9 in RCC cells after mutating the 201-252-nt region of circEHD2. **P**, Western blot assay revealed the level of SOX9 in OSRC-2 cells after mutating the 201-252-nt region of circEHD2. Error bars, standard deviation (SD) of three independent experiments. *, *P* < 0.05; **, *P* < 0.01; ***, *P* < 0.001

To explore the molecular mechanism by which circEHD2 induced progression in RCC, a biotin-coupled circEHD2 probe and control probe were used through RNA pull-down assay to identify the proteins interacting with circEHD2 in OSRC-2 and 786-O cells. (Fig. 5A). Silver staining results showed that an obvious band between 25 and 35 kDa was abundantly enriched in the circEHD2 probe group (Fig. 5B and Fig. S7A). Then, MS analysis confirmed that YWHAH was enriched in the circEHD2 probe group (Fig. 5C). Additionally, circEHD2 was validated as being specifically enriched in the circEHD2 probe group through qRT–PCR assay (Fig. 5D and Fig. S7B). Western blot of the RNA pull-down proteins showed that circEHD2 could specifically bind to YWHAH (Fig. 5E). Moreover, FISH-IF assay through confocal microscopy demonstrated that circEHD2 and YWHAH were colocalized mostly in the nucleus of OSRC-2 and 786-O cells (Fig. 5F). Consistently, the RIP assay revealed that in RCC cells, circEHD2 could be abundantly enriched by YWHAH (Fig. 5G and Fig. S7C). The sequence analysis of circEHD2 and YWHAH through NPDock (https://genesilico.pl/NPDock/) also showed that circEHD2 could bind to YWHAH (Fig. 5H). Then catRAPID (http://service.tartaglialab.com/page/catrapid_group) was used to predict the interaction region between circEDH2 and YWHAH, results showed that 201–252-nt region of circEHD2 was required for YWHAH interaction (Fig. S7D, E). Subsequently, serial deletion analysis revealed that 200–260-nt region of circEHD2 was essential for YWHAH interaction (Fig. 5I). Moreover, qRT–PCR revealed the increased level of circEHD2 following mutation of the 201–252-nt region of circEHD2 in OSRC-2 and 786-O cells (Fig. 5J), while RIP assays revealed the decreased enrichment of circEHD2 by YWHAH antibodies following the mutation (Fig. 5K and Fig. S7F). These data suggest that the 201–252-nt region of circEHD2 was crucial for the YWHAH interaction.

### circEHD2 and YWHAH interaction is crucial for RCC progression

To estimate the vital role of circEHD2 binding to YWHAH in the progression of RCC, we first designed two si-RNAs that specifically target YWHAH. The results showed that YWHAH was significantly inhibited when transfected with si-YWHAHs (Fig. 5L). However, we found that the mRNA level of YWHAH was not influenced by the downregulation of circEHD2 (Fig. S7G). Then, qRT–PCR showed that SOX9 was downregulated when transfected with si-YWHAHs in OSRC-2 and 786-O cells (Fig. 5M). Similar results were obtained by western blot assays (Fig. 5N and Fig. S7H). In addition, we found that the expression of circEHD2 was not affected by the knockdown of YWHAH (Fig. S7I). Next, we observed a significant decrease in the expression level of SOX9 following the mutation of the 201–252-nt region of circEHD2, as confirmed by qRT–PCR and western blot assays (Fig. 5O, P and Fig. S7J). These data confirm that the interaction between circEHD2 and YWHAH is important for the progression of RCC.

### YWHAH directly interacts with YAP in RCC

**Fig. 6:**
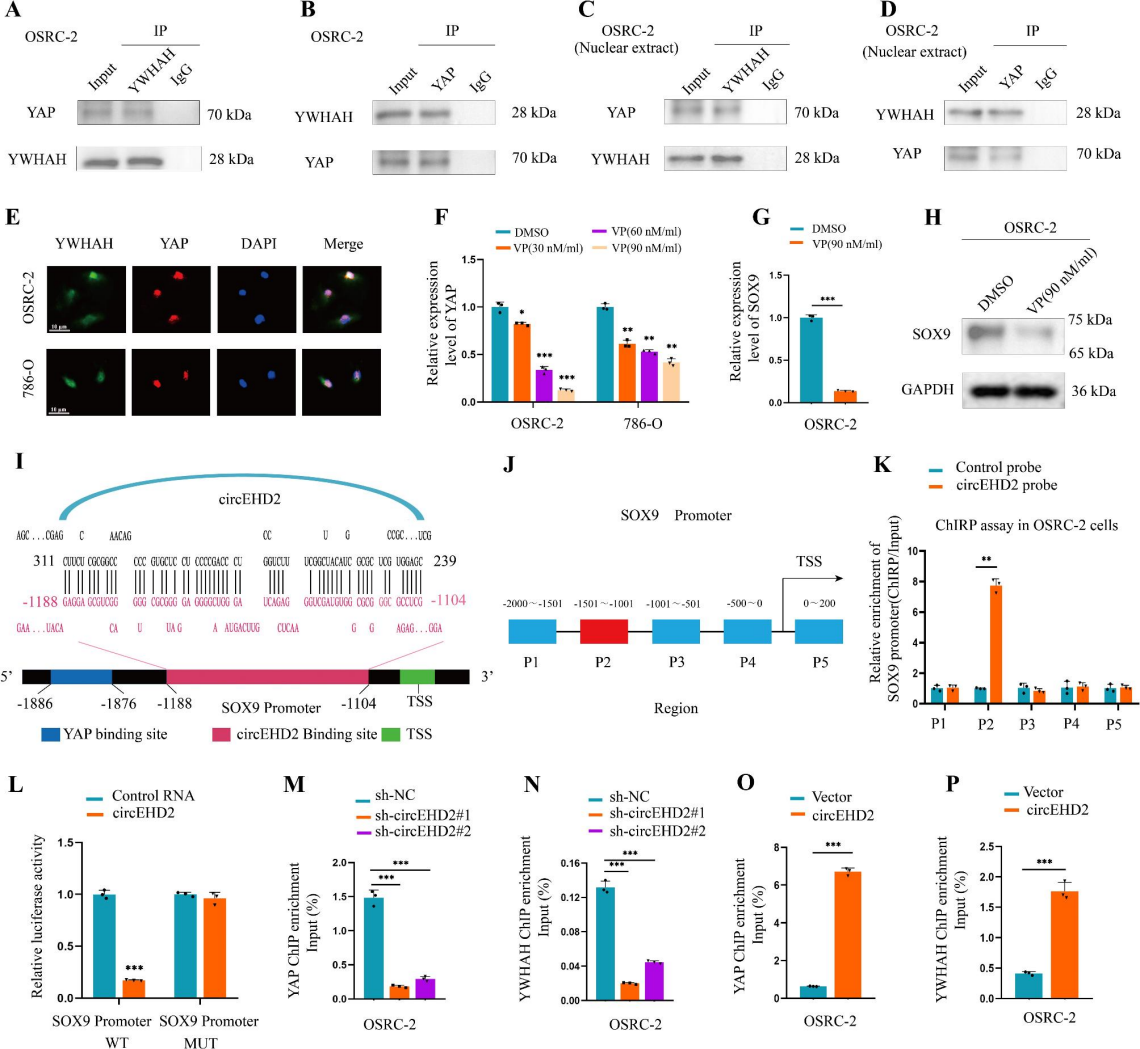
YWHAH recruits circEHD2 and YWHAH to the promoter of SOX9. **A** and **B**, Co-IP assay showed that YAP could be precipitated by anti-YWHAH antibody (**A**) and YWHAH could be precipitated by anti-YAP antibody (**B**) in OSRC-2 cells. **C** and **D**, Co-IP assay confirmed that YWHAH could bind to YAP by anti-YWHAH antibody (**C**) and anti-YAP antibody (**D**) in the nuclear extracts of OSRC-2 cells. **E**, Subcellular co-localization of YWHAH and YAP in RCC cells was measured by fluorescence staining assay. Scale bars: 10 μm. **F**, qRT–PCR analysis of the level of YAP in RCC cells when cocultured with VP at different doses. **G** and **H**, qRT–PCR analysis (**G**) and western blot assay (**H)** showed the expression level of SOX9 in OSRC-2 cells when cocultured with VP. **I**, Schematic illustration of the binding sites of circEHD2 and YAP on SOX9 promoter. **J**, Schematic illustration of PCR-amplified fragments of SOX9 promoter. **K**, ChIRP analysis the enrichment of SOX9 promoter fragments in OSRC-2 cells. **L**, Dual-luciferase reporter assays showed that circEHD2 could bind to the P2 region (-1501-nt to -1001-nt) of the SOX9 promoter. **M–P**, ChIP-qPCR of YAP (**M** and **O**) and YWHAH (**N** and **P**) revealed the enrichment of SOX9 promoter in OSRC-2 cells with circEHD2 knockdown (**M** and **N**) or overexpression (**O** and **P**). Error bars represent the standard deviation (SD) of three independent experiments. *, *P* < 0.05; **, *P* < 0.01; ***, *P* < 0.001

YWHAH (14-3-3η), a member of the YWHA (or 14-3-3) family, usually functions by interacting with other proteins to mediate cellular functions, including those in cancer [27–29]. Research has shown that YWHAH can bind to YAP to regulate the downstream genes [27]. To verify whether YWHAH plays a vital role in RCC by interacting with YAP, NPDock (https://genesilico.pl/NPDock/) shows that YWHAH could bind perfectly to YAP (Fig. S8A). To validate this hypothesis, Co-IP assays were performed in OSRC-2 and 786-O cells with anti-YWHAH antibodies and anti-YAP antibodies. The results revealed that YWHAH bound directly to YAP (Fig. 6A, B and Fig. S8B, C). As our previous experiments revealed that circEHD2 and YWHAH were mostly colocalized in the nucleus, Co-IP assays were conducted using the nuclear extracts of OSRC-2 and 786-O cells with anti-YWHAH and anti-YAP antibodies, the results were consistent with those observed in the whole-cell protein extracts (Fig. 6C, D and Fig. S8D, E). Furthermore, the FISH-IF assay indicated that YWHAH and YAP were colocalized mostly in the nucleus of RCC cells by confocal microscopy (Fig. 6E). YAP, as a core transcriptional regulator, is involved in cell proliferation, tissue homeostasis, apoptosis, and invasion of human malignancies [30, 31]. Verteporfin (VP), as an inhibitor of YAP, has been proven to inhibit YAP expression by disturbing the interaction between YAP and TEAD in several human malignancies [30, 32, 33]. Then, we found that the expression level of YAP was downregulation when cocultured with VP at different doses (Fig. 6F). Similarly, we found a decreased level of SOX9 in OSRC-2 and 786-O cells when cocultured with VP by qRT–PCR and western blot assays (Fig. 6G, H and Fig. S8F, G). Our findings demonstrated that YWHAH could interact with YAP in the nucleus of RCC cells.

### YWHAH acts as a bridge to recruit circEHD2 and YAP to the promoter of SOX9

In order to explore how circEHD2 regulates the expression of SOX9 through YWHAH and YAP in RCC cells, we used the RNAInter platform (http://www.rna-society.org/rnainter/) to predict the relationship between circEHD2 and the SOX9 promoter, and between YAP and the SOX9 promoter. The results showed that circEHD2 directly interacted with the − 1188-nt to -1104-nt region (referred to as P2) of the SOX9 promoter, while YAP directly bound to the − 1886-nt to -1876-nt region (referred to as P1) of the SOX9 promoter (Fig. 6I). Next, chromatin isolation by ChIRP assay revealed that the P2 region (-1501-nt to -1001-nt) of the SOX9 promoter was the interaction region for circEHD2 (Fig. 6J, K and Fig. S8H). Then, we constructed luciferase plasmids containing the wild-type and mutant (-1188-nt to -1104-nt) versions of the SOX9 promoter. The luciferase reporter analyses indicated that circEHD2 obviously reduced the luciferase activity when co-transfected with the wild SOX9 promoter plasmid, while the reverse results were obtained when co-transfected with the SOX9-mutation promoter plasmid (Fig. 6L). We also found a decreasing enrichment of the SOX9 promoter by YAP following knockdown of circEHD2 by ChIP assay, as well as an increased enrichment of the SOX9 promoter by YAP when circEHD2 was upregulated via the ChIP assay (Fig. 6M, O and Fig. S8I, K). Similar results were observed when the YWHAH antibody was used in the ChIP assays (Fig. 6N, P and Fig. S8J, L). Taken together, our results confirm that YWHAH works as a bridge to recruit circEHD2 and YAP to the promoter of SOX9 in RCC.

### YWHAH and YAP partially reverse the oncogenic effects induced by circEHD2

To determine the role of YWHAH and YAP in the circEHD2-induced progression of RCC, rescue experiments were applied in OSRC-2 and 786-O cells. First, western blotting showed a decreasing level of SOX9 following YWHAH knockdown in OSRC-2 cells with circEHD2 overexpression (Fig. S9A). Next, as shown by the results of EdU assay and transwell assays, the proliferation, invasion, and migration abilities of OSRC-2 were significantly inhibited in circEHD2-overexpressing OSRC-2 cells when transfecting with si-YWHAH#1 (Fig. S9B–F). Additionally, the expression level of SOX9 was downregulated in 786-O cells with circEHD2-overexpression after coculturing with Verteporfin (VP) (Fig. S10A). Moreover, the results of EdU and transwell assays demonstrated that the proliferation, invasion, and migration abilities of 786-O cells with circEHD2-overexpression were significantly inhibited when cocultured with VP (Fig. S10B–F). Therefore, our results illustrate that YWHAH and YAP could partially reverse the tumor-promoting effects induced by circEHD2 in RCC.

### circEHD2 induced the upregulation of SOX9, boosting the progression of RCC

Next, we clarified the vital role of SOX9 in the progression of RCC mediated by circEHD2. Our previous results showed that the expression level of SOX9 was in accordance with upregulation or downregulation of circEHD2 in RCC cells (Fig. 4D, E). Then, we found that the si-SOX9s could efficiently inhibit SOX9 expression, while the overexpression plasmid could significantly increase SOX9 expression (Fig. S11A, B). However, the expression of circEHD2 was not influenced by the downregulation or upregulation of SOX9 (Fig. S11C, D). Next, EdU assay and transwell assay showed that SOX9 treatment rescued the inhibitory effects of proliferation, invasion, and migration induced by the knockdown of circEHD2 in OSRC-2 cells (Fig. S12A–E). Additionally, we found that silencing SOX9 could significantly inhibit the growth, invasion, and migration of 786-O cells with circEHD2-overexpression through EdU assay and transwell assay (Fig. S12F–J). These data provide further evidence that circEHD2-mediated SOX9 expression induces the progression of RCC.

### FUS promotes the biogenesis of circEHD2 in RCC

**Fig. 7:**
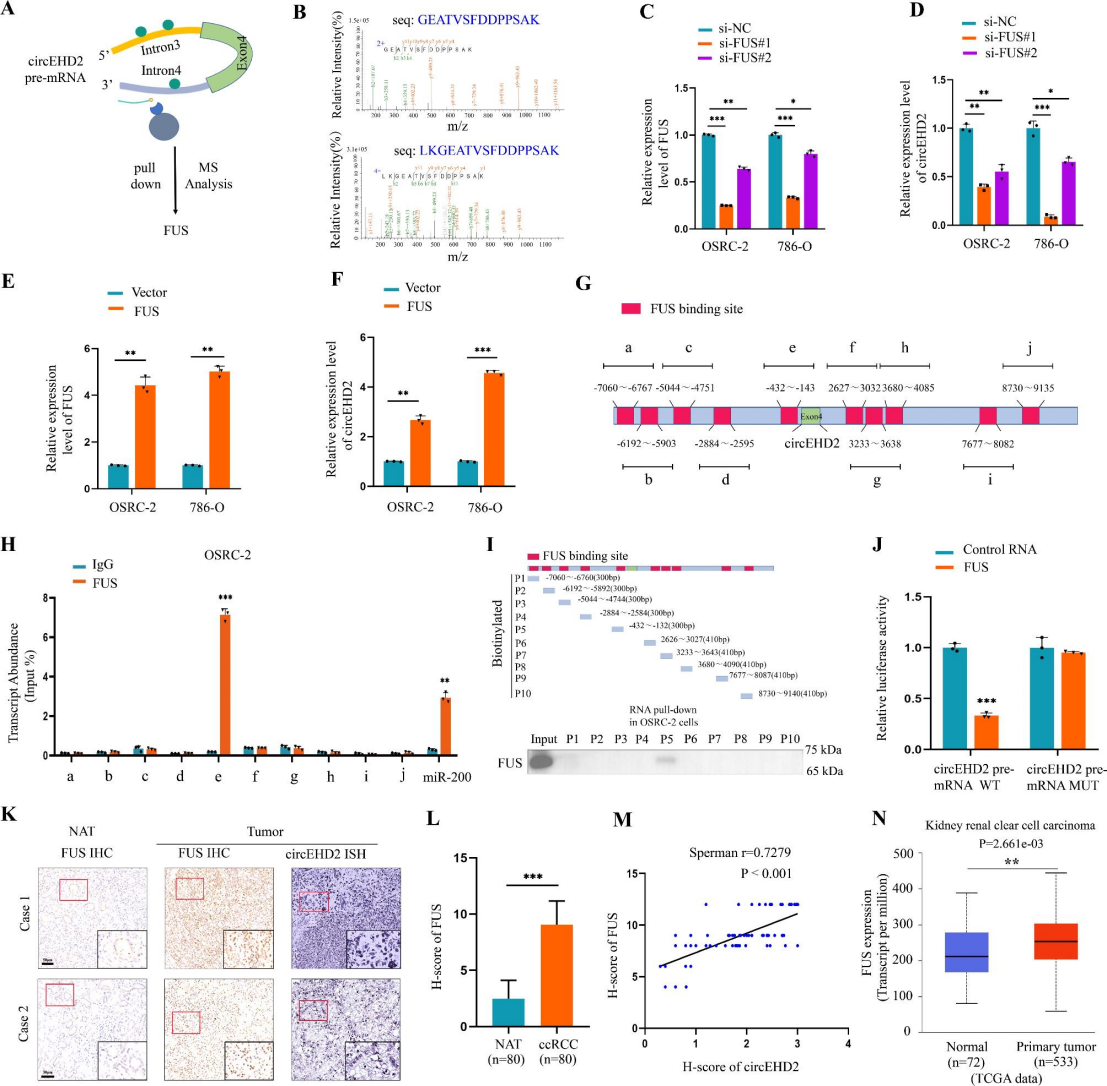
FUS promotes the biogenesis of circEHD2. **A**, Schematic illustration of circEHD2 pre-mRNA pull-down following mass spectrometry (MS) to identify circEHD2 splicing factors. **B**, MS analysis of splicing factors that interacted with circEHD2 pre-mRNA via RNA pull-down assay. **C** and **D**, qRT–PCR analysis of the level of FUS and circEHD2 in RCC cells when transfected with si-FUS. **E** and **F**, qRT–PCR analysis the level of FUS and circEHD2 in RCC cells when overexpression of FUS. **G**, The putative binding sites of FUS in the upstream and downstream of circEHD2 pre-mRNA were predicted by catRAPID. **H**, The RIP assay was performed to confirm the binding sites of FUS on circEHD2 pre-mRNA in OSRC-2 cells. miR-200 was used as the positive control. **I**, The RNA pull-down assay analyzed the interaction between FUS and above 10 fragments of circEHD2 pre-mRNA (P1-P10) in OSRC-2 cells. **J**, Dual-luciferase reporter assays showed that FUS could bind to circEHD2 pre-mRNA (-432-nt to -143-nt). **K**, Representative images of IHC and ISH showed the expression of FUS and circEHD2 in ccRCC tissues and paired NATs (n = 80). Scale bars: 50 μm. **L**, The H-score of FUS in ccRCC tissues and paired NATs (n = 80). **M**, The Pearson correlation analysis showed that the level of FUS was positively correlated with that of circEHD2. **N**, The level of FUS was upregulated in kidney renal clear cell carcinoma from the TCGA database. Error bars represent the standard deviation (SD) of three independent experiments. *, *P* < 0.05; **, *P* < 0.01; ***, *P* < 0.001

As previously reported, RNA-binding proteins could regulate the biogenesis of circRNAs post-transcriptionally [10, 34–36]. To investigate the potential splicing factors involved in the cyclization of circEHD2, an RNA pull-down assay targeting the pre-mRNA of circEHD2 (in vitro transcription) was conducted in OSRC-2 cells, and MS analysis revealed that FUS was the only splicing factor that interacted with circEHD2 pre-mRNA (Fig. 7A, B). To verify the regulatory effects of FUS on circEHD2 in RCC cells, the knockdown and overexpression of FUS in OSRC-2 and 786-O cells were established (Fig. 7C, E). Then, the results of qRT–PCR indicated that the expression level of circEHD2 was in accordance with that of FUS (Fig. 7D, F). However, the level of FUS was not influenced by the downregulation or upregulation of circEHD2 (Fig. S13A, B).

We further examined the direct binding sites of FUS on circEHD2 pre-mRNA. First, catRAPID (http://service.tartaglialab.com/page/catrapid_group) showed 10 putative binding sites in the upstream and downstream of circEHD2 pre-mRNA (Fig. 7G). Then, RIP assay showed that FUS was only intensely related to the putative binding sites (referred to as site “e”) upstream of circEHD2 pre-mRNA (Fig. 7H and Fig. S13C). Next, RNA pull-down assays also demonstrated that FUS could interact with the “P5” fragment of circEHD2 pre-mRNA using an in vitro transcript of the putative RNA fragments (Fig. 7I and Fig. S13D). Additionally, the results of the luciferase reporter assay confirmed that FUS could bind to the site “e” of circEHD2 pre-mRNA when FUS mRNA was co-transfected with the wild and mutated circEHD2 pre-mRNA (-432-nt to -143-nt) (Fig. 7J). Moreover, IHC showed that FUS was upregulated in ccRCC tissues compared to the paired NATs (Fig. 7K, L), and a positive correlation was noted between FUS and circEHD2 (Fig. 7M). Furthermore, we found a high expression level of FUS in kidney renal clear cell carcinoma according to TCGA database (Fig. 7N). Similar results were obtained for most types of human cancer via TCGA database (Fig. S13E). Collectively, our results suggest that the splicing factor FUS participates in the biogenesis of circEHD2 in RCC.

### hnRNPA2B1 regulated circEHD2 packaging into EVs

**Fig. 8:**
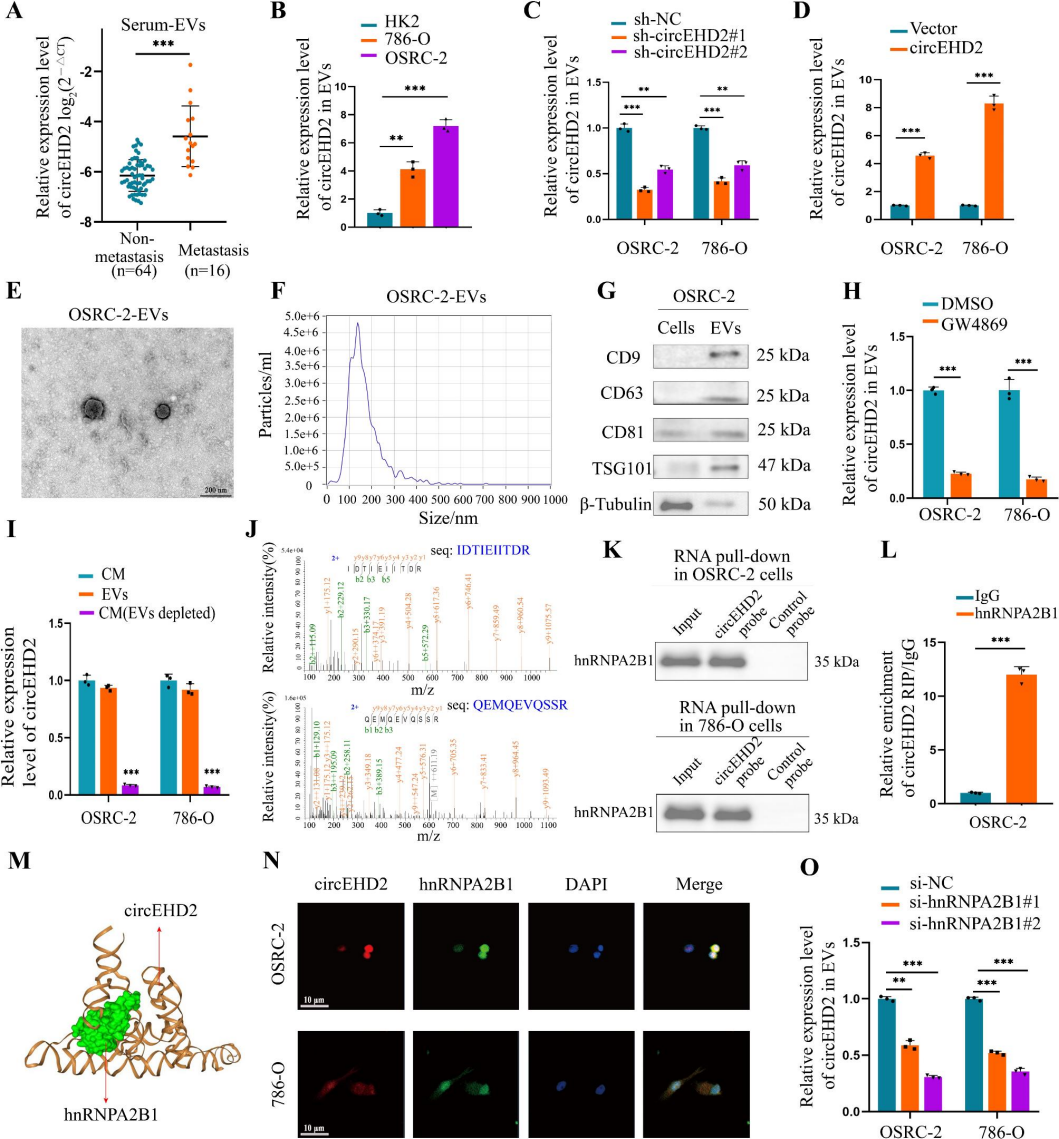
hnRNPA2B1 mediates the packaging of circEHD2 into EVs. **A**, The expression level of circEHD2 in serum EVs from RCC patients based on the metastasis status. **B**, qRT-PCR analysis of the expression level of EVs-circEHD2 in OSRC-2, 786-O, and HK2 cells. **C**, The expression level of EVs-circEHD2 in RCC cells after knockdown of circEHD2. **D**, The expression level of EVs-circEHD2 in RCC cells transfected with circEHD2. **E** and **F**, Transmission electron microscopy (TEM) and NanoSight were used to characterize the purified EVs from OSRC-2 cells. Scale bars: 200 nm. **G**, Western blot analysis of EVs markers from OSRC-2 EVs or cell lysates. **H**, qRT-PCR analysis of the expression level of circEHD2 in EVs from RCC cells treatment with GW4869 (an inhibitor of EVs secretion). **I**, qRT-PCR analysis of the expression level of circEHD2 in the CM of RCC cells after depletion of EVs by ultracentrifugation. **J**, Mass spectrometry (MS) analysis of the proteins interacting with circEHD2 via RNA pull-down assay. **K**, Western blot assay confirming the interaction between circEHD2 and hnRNPA2B1 in circEHD2 pull-down proteins. **L**, RIP assay in OSRC-2 cells confirmed that circEHD2 could be enriched by hnRNPA2B1. **M**, The three-dimensional structure showed that circEHD2 could interact with hnRNPA2B1. **N**, Subcellular co-localization of circEHD2 and hnRNPA2B1 in RCC cells measured by fluorescence staining assay. Scale bars: 10 μm. **O**, qRT-PCR analysis the expression level of EVs-circEHD2 after knockdown of hnRNPA2B1 in RCC cells. Error bars represent the standard deviation (SD) of three independent experiments. **, *P* < 0.01; ***, *P* < 0.001

Previous studies showed that EVs were involved in the metastasis of various tumors [19, 22, 23]. First, qRT-PCR indicated that circEHD2 was upregulated in the serum EVs obtained from metastasis RCC patients when compared to non-metastasis RCC patients (Fig. 8A). Subsequently, we found increased EVs-circEHD2 in RCC cell lines compared to HK2 cells (Fig. 8B). Interestingly, the enrichment of circEHD2 in EVs was closely relative to its expression in RCC cells (Fig. 8C, D), indicating that EVs-circEHD2 may play a vital role in the metastasis of RCC. In addition, classic characteristics of EVs were observed in the EVs derived from OSRC-2 and 786-O cell lines: a typical cup-shaped morphology, approximately 30–150 nm in size, and typical protein markers CD9, CD63, CD81, and TSG101 (Fig. 8E–G and Fig. S14A–C). To verify the existence of extracellular circEHD2 mainly in the form of EVs, GW4869 was used to inhibit EVs secretion. The results showed that the level of EVs-circEHD2 was significantly downregulated after treatment with GW4869, while no effects on the level of circEHD2 in RCC cells (Fig. 8H and Fig. S14D). Additionally, we found the level of circEHD2 in the culture medium of RCC cells rarely changed after treatment with RNase A, while significantly decreased after treatment with RNase A plus Triton X-100 (Fig. S14E). Notably, qRT-PCR was performed to detect the level of circEHD2 in the culture medium, EVs, and EVs-depleted culture medium (purified by ultracentrifugation). Results showed that the level of circEHD2 was significantly lower in the EVs-depleted culture medium than in EVs and total culture medium (Fig. 8I). Taken together, these results indicate that the extracellular form of circEHD2 mainly exists in EVs.

We then explore the mechanism by which circEHD2 was selectively packaged into EVs. First, RNA pull-down through a biotin-coupled circEHD2 probe was performed in OSRC-2 cells. MS analysis confirmed that hnRNPA2B1 was enriched in the circEHD2 probe group (Fig. 8J). Then, western blot of RNA pull-down proteins and RIP assay also confirmed the interaction between circEHD2 and hnRNPA2B1 (Fig. 8K, L and Fig. S14F). Additionally, the sequence analysis of circEHD2 and hnRNPA2B1 through NPDock (https://genesilico.pl/NPDock/) also showed that circEHD2 could bind to hnRNPA2B1 (Fig. 8M). Notably, FISH-IF assay demonstrated that circEDH2 and hnRNPA2B1 could be colocalized in the cytoplasm of RCC cells (Fig. 8N). As previously reported, hnRNPA2B1 could regulate the packaging of RNAs into EVs by recognizing the specific motifs GGAG/CCCU [37]. In particular, we recognized the GGAG motif in the sequence of circEHD2. Moreover, we found that EVs-circEHD2 was downregulated after knockdown of hnRNPA2B1, while the level of circEHD2 in RCC cells was not significantly affected (Fig. 8O and Fig. S14G, H). These results revealed that circEHD2 could package into EVs in an hnRNPA2B1-dependent manner.

### RCC cell derived EVs regulate fibroblasts activation

**Fig. 9:**
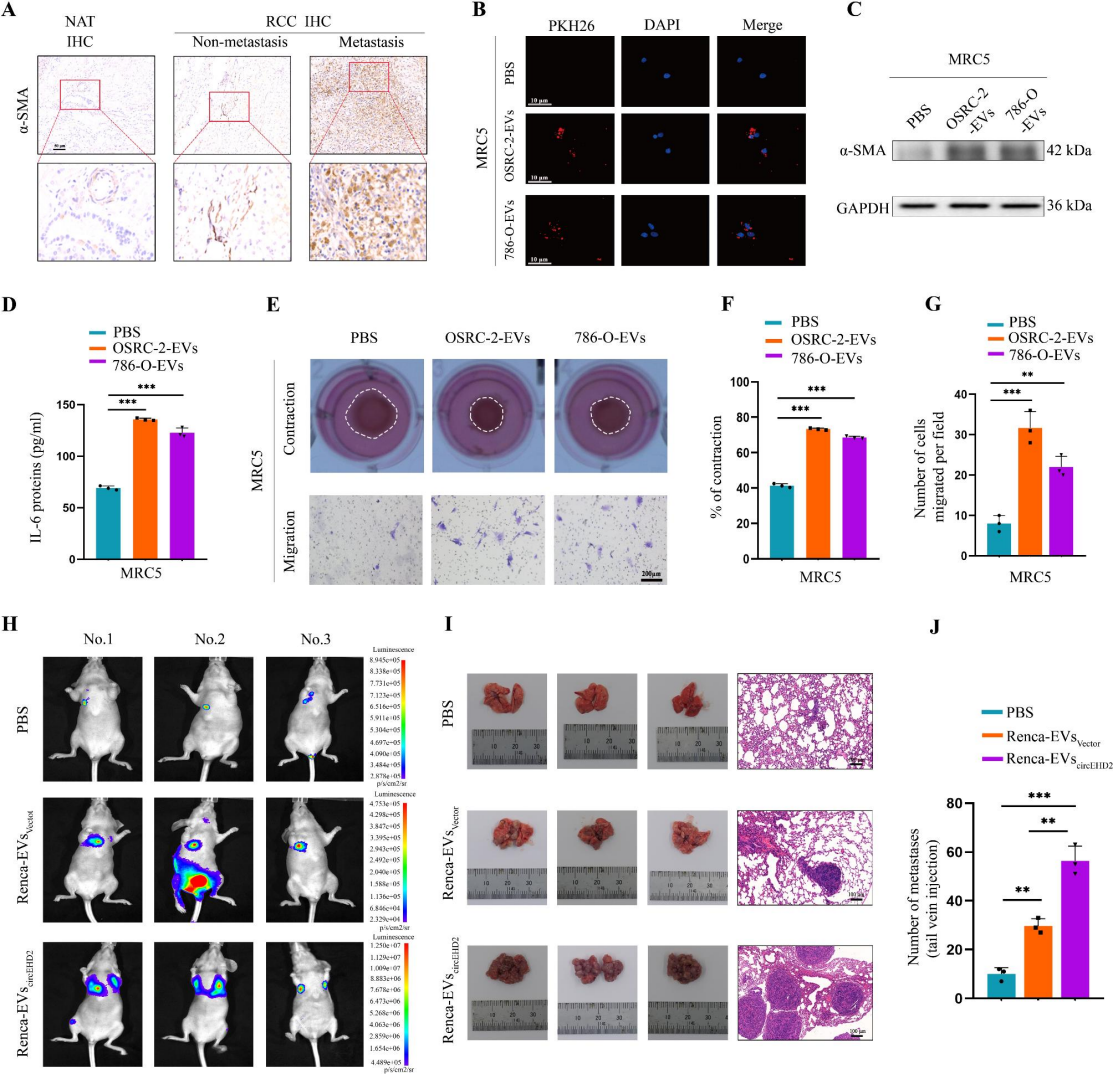
EVs-circEHD2 promotes the metastasis of RCC by regulating fibroblast activation. **A**, Representative images of α-SMA in RCC tissues, measured by IHC, according to the metastasis status. Scale bars: 50 μm. **B**, Representative images of MRC5 cells after incubation with PKH26-labeled RCC-EVs. Scale bars: 10 μm. **C**, Western blot showing the level of α-SMA in MRC5 cells after incubation with RCC-RVs. **D**, The ELISA results of IL-6 in MRC5 cells after incubation with RCC-EVs. **E**-**G**, Representative images of MRC5 cells cultured with PBS, OSRC-2-EVs and 786-O-EVs. Scale bars: 200 μm. (**F**) The collagen contraction and (**G**) the number of migrated cells. **H-J**, Representative images of in vivo bioluminescence imaging (**H**), gross appearance of the lung (**I**, left), hematoxylin and eosin staining (HE) (**I**, right), and quantification of lung metastatic colonization (**J**) in the tail vein of lung metastasis model treated with PBS (n = 3/group), Renca-EVs_Vector_ (n = 3/group), and Renca-EVs_circEHD2_ (n = 3/group). Error bars represent the standard deviation (SD) of three independent experiments. **, *P* < 0.01; ***, *P* < 0.001

CAFs were activated fibroblasts in tumor tissues and were demonstrated to be involved in metastasis by releasing various cytokines, including IL-6 [16–18]. We first verified the expression of α-SMA in RCC tissues, which is the most effective marker of CAFs. Results showed a high level of α-SMA in RCC tissues with metastasis when compared to non-metastasis RCC tissues by IHC (Fig. 9A). To investigate whether RCC cells derived EVs-circEHD2 could activate fibroblasts, a coculture system with MRC5 cells was established. First, EVs were purified from RCC cells and labeled with PKH26(red), then cocultured with MRC5 cells for 24 h. After incubation, the red fluorescence signal was observed in the cytoplasm of recipient MRC5 cells, indicating that EVs secreted by RCC cells could deliver to MRC5 cells (Fig. 9B). Subsequently, we found the level of α-SMA was upregulated in MRC5 cells after incubation with EVs-circEHD2 derived from RCC cells (Fig. 9C). More importantly, fibroblasts incubated with EVs derived from RCC cells showed a higher level of IL-6, which has an important role in promoting carcinoma metastasis (Fig. 9D). As activated fibroblasts have the enhanced ability of migration and matrix adhesions [16, 17], we investigated the effects of RCC cells derived EVs-circEHD2 on fibroblast-mediated migration and collagen contraction. As compared to the control group, MRC5 incubated with EVs derived from RCC cells displayed enhanced migration and contraction abilities (Fig. 9E–G). Furthermore, the migration and contraction abilities of MRC5 could be dramatically facilitated after incubation with EVs secreted by circEHD2-overexpression 786-O cells (Fig. S15A–C). Conversely, EVs derived by OSRC-2 cells with circEHD2 knockdown could abolish the migration and contraction abilities of MRC5 cells (Fig. S15D–F). These results suggest that EVs-circEHD2 derived from RCC cells induced fibroblast activation.

### EVs-circEHD2 promoted metastasis in vivo

To investigate whether RCC derived EVs-circEHD2 promotes metastasis in vivo, a tail vein lung metastasis model was established. First, we found that the sequence of mouse circEhd2 exhibited high conservation with human circEHD2, with more than 89% homology to its human ortholog (Fig. S16A). Additionally, qRT–PCR revealed that the overexpression vector of circEhd2 could effectively increase the level of circEhd2 in mouse Renca cells (Fig. S16B). Similar results were obtained in their corresponding EVs (Fig. S16C). To confirm the metastasis ability of EVs-circEhd2 in vivo, Renca cells stably expressing firefly luciferase were injected into the tail vein of nude mice, then the mice were randomly divided into three groups (n = 3/group). Then PBS (control), EVs derived from Renca cells transfected with vector (Renca-EVs_Vector_), and EVs derived from Renca cells transfected with circEHD2 (Renca-EVs_circEHD2_) were tail vein injected every three days for four consecutive weeks. The results showed that Renca-EVs_circEHD2_ could significantly promote the ability of lung metastasis of Renca cells when compared with the PBS group and Renca-EVs_Vector_ group (Fig. 9H–J). Furthermore, CAFs were demonstrated to be largely recruited in the lung metastasis induced by Renca-EVs, as verified by the level of α-SMA through IHC assays (Fig. S16D). Collectively, these results suggest that EVs-circEHD2 significantly facilitates the metastasis of RCC in vivo.

### circEHD2-ASO acts as a treatment to inhibit the growth of RCC in vivo

**Fig. 10:**
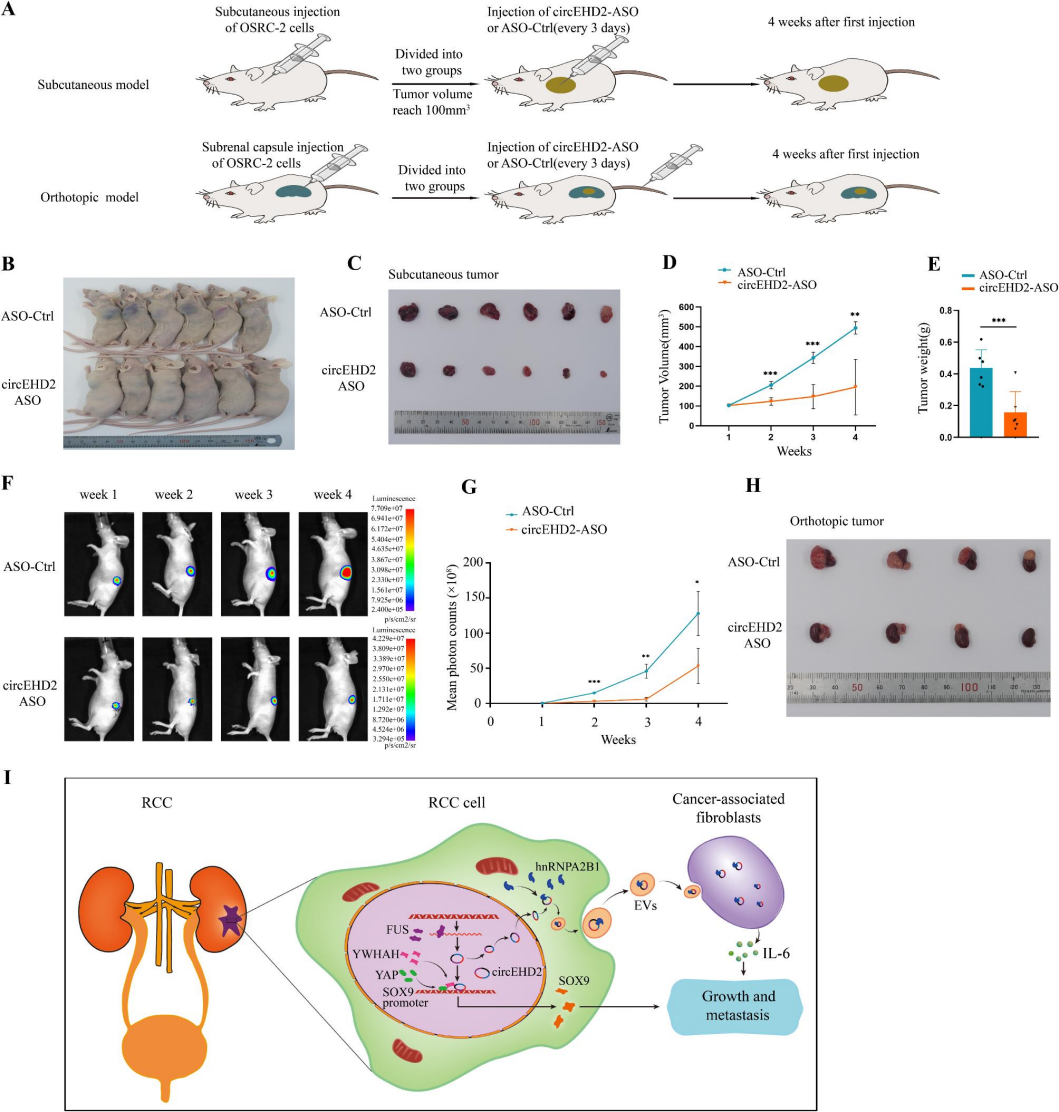
circEHD2-ASO inhibits the growth of RCC in vivo. **A**, Schematic illustration of tumor inoculation and circEHD2-ASO treatment in subcutaneous xenograft model and orthotopic xenograft model. **B–E**, Representative images of xenograft tumors (**B**), dissected tumors (**C**), the volume of tumors (**D**), and tumor weights (**E**) in subcutaneous xenograft model treated with ASO-control (n = 6/group) and circEHD2-ASO (n = 6/group), respectively. **F-H**, Representative images of in vivo bioluminescence imaging (**F**), the mean photon counts (**G**), and gross appearance of orthotopic tumor (**H**) in orthotopic xenograft model treated with ASO-control (n = 4/group) and circEHD2-ASO (n = 4/group), respectively. **I**, A schematic model showing the mechanism of EVs-circEHD2 mediated the progression of RCC. FUS mediated the biogenesis of circEHD2, then circEHD2 enhanced the growth of RCC through the circEHD2/YWHAH/YAP/SOX9 pathway. While hnRNPA2B1 mediated the packaging of circEHD2 into EVs, and EVs-circEHD2 promote metastasis of RCC by converting fibroblasts to CAFs. *, *P* < 0.05; **, *P* < 0.01; ***, *P* < 0.001

Anti-sense oligonucleotide (ASO) has been proven to be stable and effective to target and degrade target RNAs in vitro and in vivo [38–40]. Next, we validate the therapeutic potential of circEHD2 in RCC by designing circEHD2-ASO. First, qRT–PCR revealed that circEHD2-ASO effectively inhibited the expression of circEHD2 in OSRC-2 and 786-O cells (Fig. S17A). Next, FISH assay showed that circEHD2-ASO could be internalized by RCC cells (Fig. S17B). To confirm the therapeutic ability of circEHD2-ASO in vivo, a subcutaneous xenograft model and orthotopic xenograft model were established (Fig. 10A). For the therapeutic model in subcutaneous xenografts, OSRC-2 cells stably expressing firefly luciferase were implanted into the left flank of each mouse. Then, the nude mice were randomly divided into two groups (n = 6/group), which received intratumoral injections of ASO-control and circEHD2-ASO every three days. Four weeks later, the subcutaneous tumors were excised and analyzed, and the results showed that the tumor growth was significantly suppressed in the circEHD2-ASO group compared to the ASO-control group (Fig. 10B–E). Regarding the therapeutic model in orthotopic xenograft, OSRC-2 cells stably expressing firefly luciferase were implanted into the left subrenal capsule of each mouse and randomly divided into two groups (n = 4/group). Then, the circEHD2-ASO and ASO-control were injected into the tail vein every three days for four consecutive weeks. Similarly, tumor growth was clearly inhibited in the circEHD2-ASO treatment group (Fig. 10F–H). Furthermore, IHC showed that the expression levels of Ki67 and SOX9 were downregulated in the orthotopic tumor tissues treated with circEHD2-ASO (Fig. S17C–E). In conclusion, our results demonstrate that circEHD2 may serve as a potential therapeutic target in RCC, while circEHD2-ASO may provide a promising treatment method for patients with RCC.

## Discussion

In patients with RCC, fatal metachronous distant metastasis remain the major factor associated with cancer-specific mortality [41, 42]. Thus, it is urgent to investigate the underlying molecular mechanisms of RCC to identify a new and promising target for its prevention and treatment. Here, we first identified a novel circRNA-circEHD2, which was upregulated in ccRCC tissues and enriched in serum EVs of ccRCC patients with metastasis. Mechanistically, FUS regulated the biogenesis of circEHD2, then circEHD2 interacted with YWHAH in the nucleus of RCC cells, while YWHAH functioned as a bridge to recruit circEHD2 and YAP to the SOX9 promoter, thus sustaining activated SOX9 to promote RCC cell growth. Importantly, we identified that circEHD2 could be packaged into EVs by interacting with hnRNPA2B1, then EVs-circEHD2 transferred to fibroblasts and converts fibroblasts to CAFs, which ultimately promoted the metastasis of RCC. Additionally, circEHD2-ASO showed an obvious inhibition of tumor growth of RCC in vivo. These results highlight a novel mechanism by which EVs-circEHD2 mediates the progression of RCC and supports EVs-circEHD2 as a potential therapeutic target (Fig. 10I).

SOX9, as a transcription factor of the SOX family, plays a vital role in determining cell fate, cell differentiation, and tissue homeostasis in the developing and adult tissues [43]. Apart from the roles mentioned, SOX9 was validated to involve various kinds of human diseases, including cancer [43–45]. In our study, we found that SOX9 was highly expressed in ccRCC tissues and was the downstream target effector of circEHD2. However, the mechanism by which circEHD2 regulates SOX9 in RCC remains elusive. Regarding the mechanisms of action, studies have indicated that circRNAs mainly regulate the progression of RCC by sponging miRNAs [46, 47]. However, our results revealed that circEHD2 bound to YWHAH, while knockdown of YWHAH induced a decrease in SOX9. Additionally, previous studies have shown that YWHAH may function by inhibiting the translocation of YAP to the nucleus through interaction with YAP and promoting its degradation in the cytoplasm [27]. Our experiment in this study demonstrated that YWHAH mainly interacts with YAP in the nucleus of RCC cells. Verteporfin (VP), as an inhibitor of YAP, has been proven to inhibit YAP expression by disturbing the interaction between YAP and TEAD in several human malignancies [30, 32, 33]. Our results also revealed that SOX9 was significantly downregulated when cocultured with VP. We further found that both circEHD2 and YAP associated with the SOX9 promoter to upregulate SOX9 levels. More importantly, FUS was found to regulate the cyclization of circEHD2 in RCC cells. However, limited knowledge is currently available regarding the regulatory mechanisms of upstream splicing factors on circRNAs. Further research is required to elucidate the specific molecular mechanisms involved in the regulatory process. Taken together, we unveiled a novel role of circEHD2 promoting RCC growth by inducing SOX9 expression via interaction with YWHAH and YAP.

CAFs are a key component of the tumor microenvironment and play a crucial role in promoting malignant progression by secreting various types of cytokines, including IL-6 [16–18]. Meanwhile, accumulating evidence revealed that EVs are crucial mediators of intercellular communication between cancer cells and stromal cells [48]. For example, EVs-miR-1247-3p derived from liver cancer cells could be transferred to fibroblasts and convert fibroblasts to CAFs, leading to the formation of lung metastasis [49]. In colorectal cancer, the communication between colorectal cancer cells and CAFs was facilitated by EVs-miR-146a-5p and EVs-miR-155-5p, which in turn promoted the metastasis of colorectal cancer [50]. In our study, we found that EVs-circEHD2 derived from RCC cells could activate CAFs and accelerate the secretion of IL-6. However, the mechanism by which circEHD2 is encapsulated into EVs remains unknown. Studies showed that some specific RNA-binding proteins could regulate the packaging of RNAs into EVs [51]. The RNA-binding protein hnRNPA2B1 has been identified to play a role in the encapsulation of RNA into EVs by recognizing specific motifs, such as GGAG/CCCU [37]. Herein, we identified the presence of the GGAG motif within the sequence of circEHD2. Additionally, we observed a decreased expression of circEHD2 in EVs after the knockdown of hnRNPA2B1. In our study, although EVs-circEHD2 was demonstrated to activate CAFs, the exact molecular mechanisms by which circEHD2 regulated the expression of IL-6 in CAFs required further investigation. These results indicated that EVs-circEHD2 may serve as a distinctive tactic for the treatment of RCC with metastasis.

ASO has been previously demonstrated as an effective treatment to inhibit target mRNA expression in vivo, and several ASOs have been applied in clinical trials [52, 53]. Previous studies have confirmed that ASO have strong inhibition efficacy, high cellular uptake efficacy, low toxicity, and long half-life compared to other RNA interference methods such as si-RNAs [38–40, 54]. Owing to their anti-tumor role in vitro and in vivo [38, 39], ASO may represent a promising therapeutic vehicle in cancer. Our results indicate that circEHD2-ASO could effectively inhibit circEHD2 expression in vitro and in vivo; thus, circEHD2-ASO may be a novel strategy for treating RCC.

## Conclusion

In summary, we elucidated a novel mechanism by which EVs-circEHD2 induced the progression of RCC. Mechanistically, FUS mediated the biogenesis of circEHD2, while YWHAH acted as a bridge to recruit circEHD2 and YAP to the promoter of SOX9, thus sustaining the activation of SOX9 to promote the growth of RCC. More importantly, hnRNPA2B1 mediated the packaging of circEHD2 into EVs, then EVs-circEHD2 activated fibroblasts to become CAFs, thus inducing the metastasis of RCC. Our findings suggest that EVs-circEHD2 represents an encouraging diagnostic marker and therapeutic strategy for RCC.

## Electronic supplementary material

Below is the link to the electronic supplementary material.

Supplementary Material 1

## Data Availability

All the data supporting the findings of this study are available within the article and its additional files.

## Abbreviations

ccRCC: clear cell renal cell carcinoma
NATs: normal adjacent tissues
circRNA: circular RNA
circEHD2: circular RNA EHD2
YWHAH: yrosine 3-monooxygenase/tryptophan 5-monooxygenase activation protein eta
YAP: Yes1-associated transcriptional regulator
SOX9: SRY-box transcription factor 9
FUS: FUS RNA -binding protein
hnRNPA2B1: Heterogeneous nuclear ribonucleoprotein A2/B1
ASO: antisense oligonucleotides
HK2: human renal proximal tubular epithelial cell line
ATCC: American Type Culture Collection
FBS: Fetal bovine serum
PBS: phosphate buffered saline
TEM: transmission electron microscopy
gDNA: genomic DNA
cDNA: complementary DNA
qRT-PCR: Quantitative real-time PCR
WB: Western blot
MS: Mass spectrometry
HE: Hematoxylin-eosin
IHC: Immunohistochemistry
FISH: Fluorescence in situ hybridization
FISH-IF: Fluorescence in situ hybridization and Immunofluorescence
ISH: In situ hybridization
RIP: RNA immunoprecipitation
ChIP: Chromatin immunoprecipitation
ChIRP: Chromatin isolation by RNA purification
shRNA: Short hairpin RNA
si-RNA: small interfering RNA
WT: wild type
DIG: digoxin
BCIP/NBT: 5-Bromo-4-Chloro-3-Indolylphosphate/Nitroblue Tetrazolium
DAPI: 4′,6-diamidino-2-phenylindole
ROC: Receiver operating characteristic
PFS: progression-free survival
EVs: Extracellular vesicles
CAFs: Cancer-associated fibroblasts
