# Supplementary material for: Extracellular vesicle-circEHD2 promotes the progression of renal cell carcinoma by activating cancer-associated fibroblasts
Source: Mol Cancer. 2023 Jul 22;22:117. doi: 10.1186/s12943-023-01824-9 (PMC10362694; doi:10.1186/s12943-023-01824-9)
Supplement: Supplementary file 1 — Supplementary Material 1 [file 12943_2023_1824_MOESM1_ESM.docx]

**Extracellular vesicle-circEHD2 promotes the progression of renal cell carcinoma by activating cancer-associated fibroblasts**

**Supplementary Materials**

**
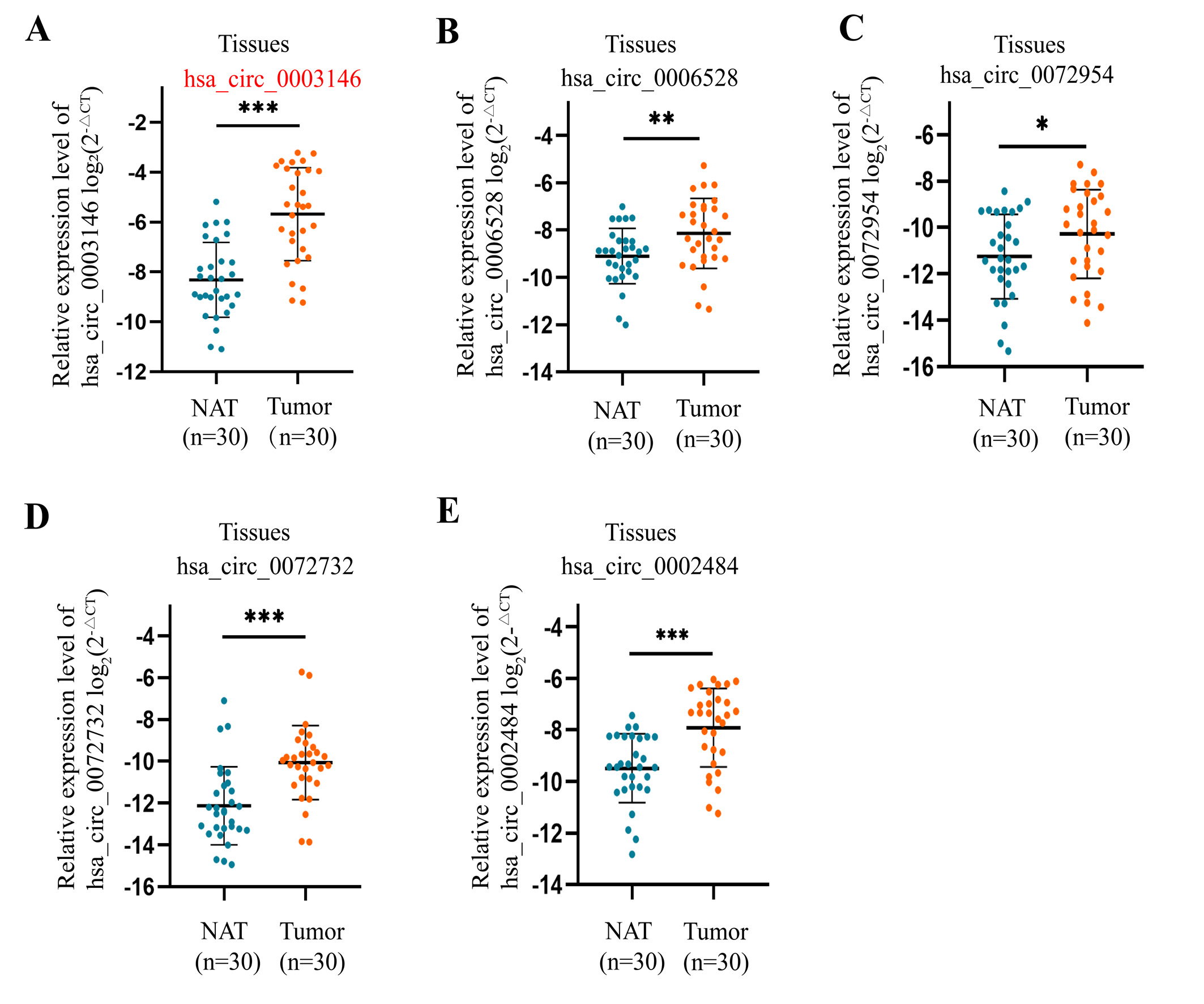
**

**Fig S1. The expression level of 5 candidate circRNAs in the ccRCC tissues and paired NATs**. (**A**–**E**) qRT-PCR analysis of the level of 5 candidate circRNAs in ccRCC tissues and paired NATs (n=30). (**A**) hsa_circ_0003146, (**B**) hsa_circ_0006528, (**C**) hsa_circ_0072954, (**D**) has_circ_0072732 and (**E**) hsa_circ_0002484. *, *P*<0.05; **, *P*<0.01; ***, *P*<0.001.


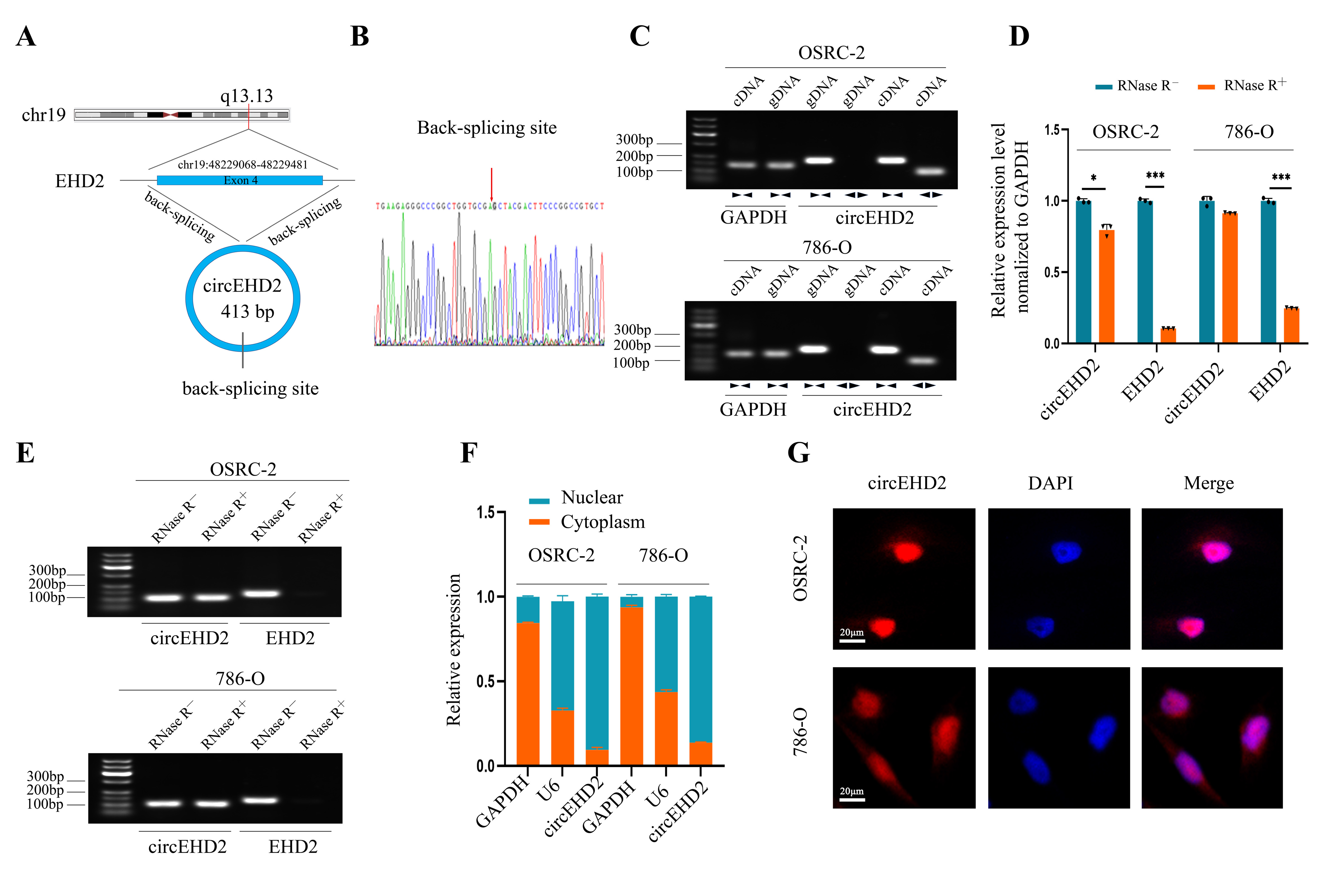


**Fig S2. The characterization of circEHD2 in RCC cells. A**, Schematic illustration revealing circEHD2 derived from exon-4 of EHD2 transcript. **B**, Rolling circle reverse transcription and Sanger sequencing confirmed the full length of circEHD2 in OSRC-2 cells. The arrow indicated the back splicing junction site of circEHD2. **C,** Convergent and divergent primers were used to amplify circEHD2 in cDNA and gDNA.circEHD2 could be amplified by divergent primers in cDNA but not in genomic DNA (gDNA). GAPDH was used as a control for a linear RNA transcript. **D** and **E,** The stability of circEHD2 and linear EHD2 was measured by PCR and qRT-PCR after treatment with RNase R in OSRC-2 and 786-O cells. **F** and **G**, The sub-cellular localization of circEHD2 in OSRC-2 and 786-O cells was determined by cytoplasmic-nuclear RNA fractionation assay and RNA FISH assay. GAPDH and U6 were used as cytoplasm control and nucleus control, respectively. Scale bars: 20 μm. Error bars, standard deviation (SD) of three independent experiments. *, *P*<0.05; ***, *P*<0.001.

**
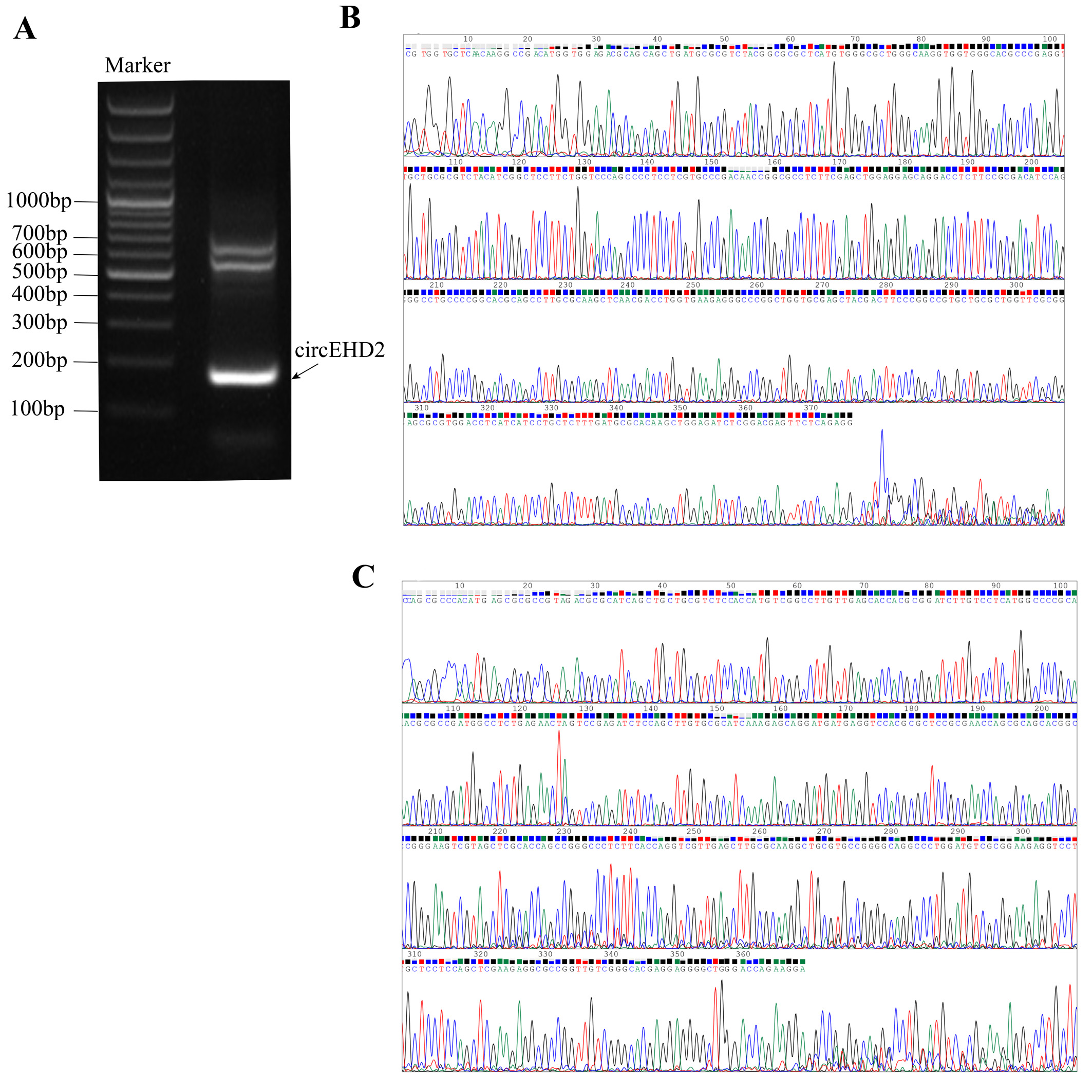
**

**Fig S3.** **Rolling circle reverse transcription and Sanger sequencing were performed to detect the full-length sequence of circEHD2 in OSRC-2 cells. A**, Image of agarose gel electrophoresis from the rolling circle reverse transcription products. **B**, The full sequence from the primer of RT1314-N1-F. **C**, The full sequence from the primer of RT1314-N1-R.

**
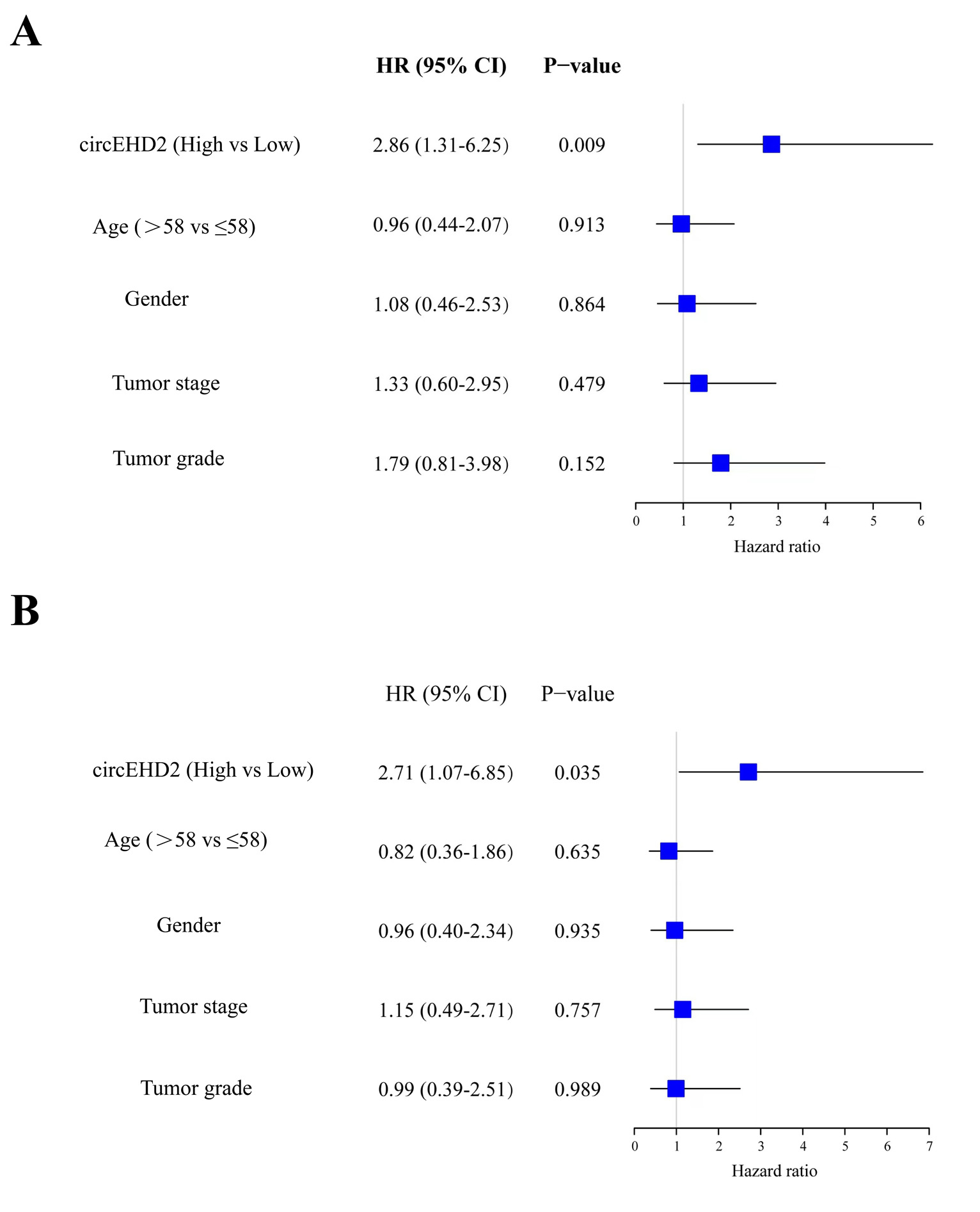
**

**Fig S4. Forest plots showing the prognostic value of tissue-circEHD2 in ccRCC patients (n = 80).** **A**, Univariate analysis of the prognostic value for tissue-circEHD2 expression in ccRCC patients (n = 80). **B,** Multivariate analysis of the prognostic value for tissue-circEHD2 expression in ccRCC patients (n = 80).


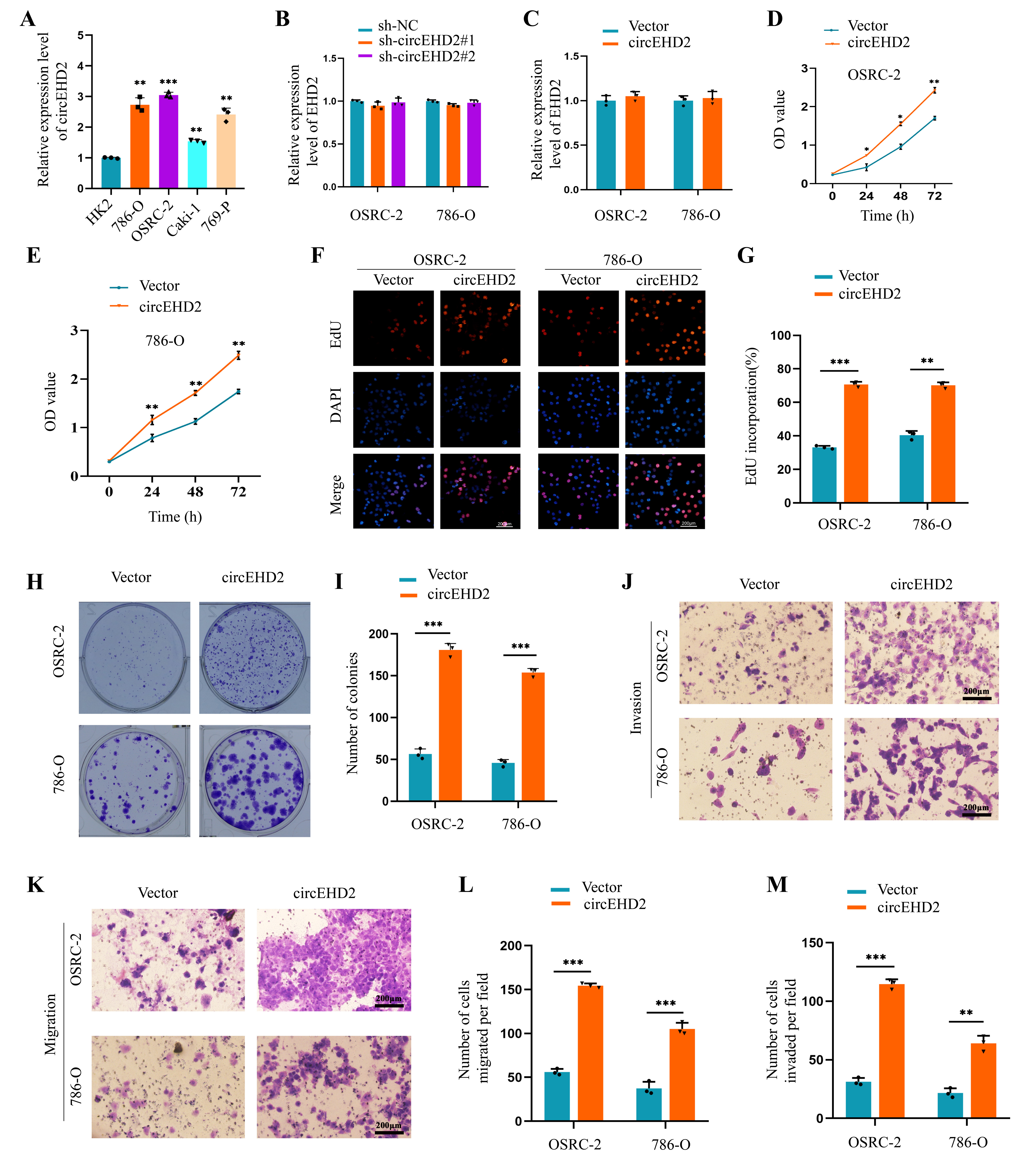


**Fig S5. Upregulation of circEHD2 enhances the proliferation, invasion, and migration of RCC cells. A**, qRT-PCR analysis the expression level of circEHD2 in 4 human RCC cell lines and one human renal proximal tubular epithelial (HK2) cell. **B** and **C**, qRT-PCR analysis of the level of EHD2 mRNA after knockdown or overexpress of circEHD2 in OSRC-2 and 786-O cells. **D** and **E**, The CCK-8 assays were used to measure the proliferation ability of OSRC-2 (**D**) and 786-O cells (**E**) with circEHD2 overexpression. **F** and **G**, EdU assay showed that overexpression of circEHD2 could accelerate the proliferation ability of OSRC-2 and 786-O cells. Scale bars: 200μm. **H** and **I**, The colony formation assay revealed the colony formation ability of OSRC-2 and 786-O cells was increased after transfecting with circEHD2. **J** and **M**, Transwell invasion assay was used to measure the invasion ability of OSRC-2 and 786-O cells after overexpression of circEHD2. Scale bars: 200μm. **K** and **L**, Transwell migration assay was carried out to assess the migration ability of OSRC-2 and 786-O cells after overexpression of circEHD2. Scale bars: 200μm. Error bars, standard deviation (SD) of three independent experiments. *, *P*<0.05; **, *P*<0.01; ***, *P*<0.001.


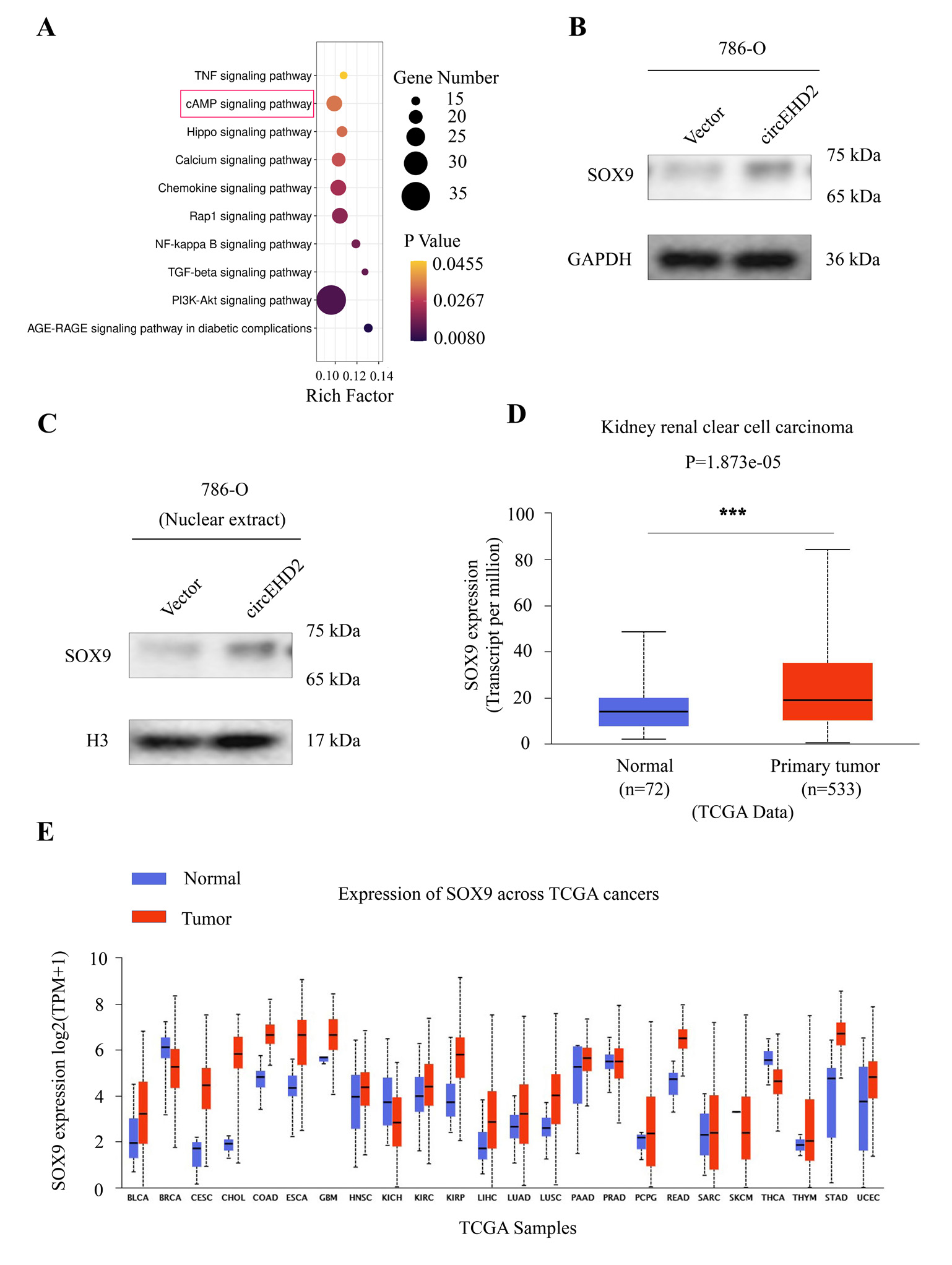


**Fig S6. SOX9 is the target gene of circEHD2.** **A**, KEGG pathway enrichment analysis revealed the enriched pathways in OSRC-2 cells with circEHD2 knockdown. **B** and **C**, Western blot showed the expression level of SOX9 in whole protein extracts (**B**) and nuclear protein extracts (**C**) of 786-O cells with overexpression of circEHD2. **D**, The level of SOX9 was upregulated in kidney renal clear cell carcinoma from the TCGA database. **E**, SOX9 was highly expressed in most cancers from the TCGA database. Error bars, standard deviation (SD) of three independent experiments. ***, *P*<0.001.


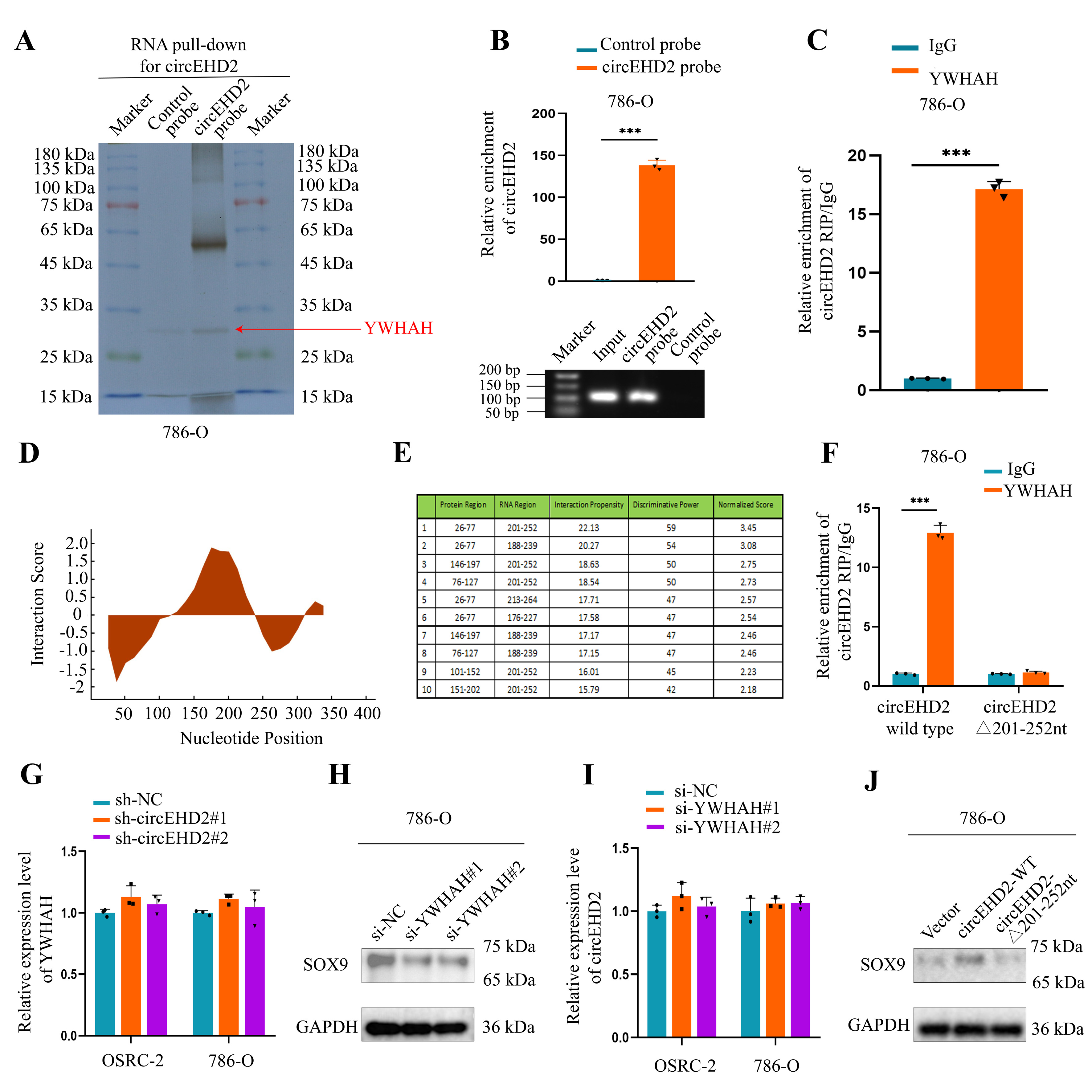


**Fig S7. circEHD2 can bind to YWHAH. A**, The silver staining image of RNA pull-down with circEHD2 probe in 786-O cells. **B**, qRT–PCR analysis confirmed that circEHD2 could be enriched by circEHD2 probe in 786-O cells by RNA pull-down assay. **C**, RIP assay in 786-O cells confirmed that circEHD2 could be enriched by YWHAH. IgG was used as a negative control. **D** and **E**, The catRAPID was used to predict the binding site of circEHD2 to YWHAH. **F**, RIP assay was conducted in 786-O cells after mutating the 201-252-nt region of circEHD2. **G**, qRT–PCR analysis of the level of YWHAH after knockdown of circEHD2 in OSRC-2 and 786-O cells. **H**, Western blot analysis of the expression of SOX9 when downregulation of YWHAH in 786-O cells. **I**, qRT–PCR analysis of the level of circEHD2 after downregulation of YWHAH in OSRC-2 and 786-O cells. **J**, Western blot assay showed a decreased level of SOX9 in 786-O cells after mutating the 201-252-nt region of circEHD2. Error bars, standard deviation (SD) of three independent experiments. ***, *P*<0.001.


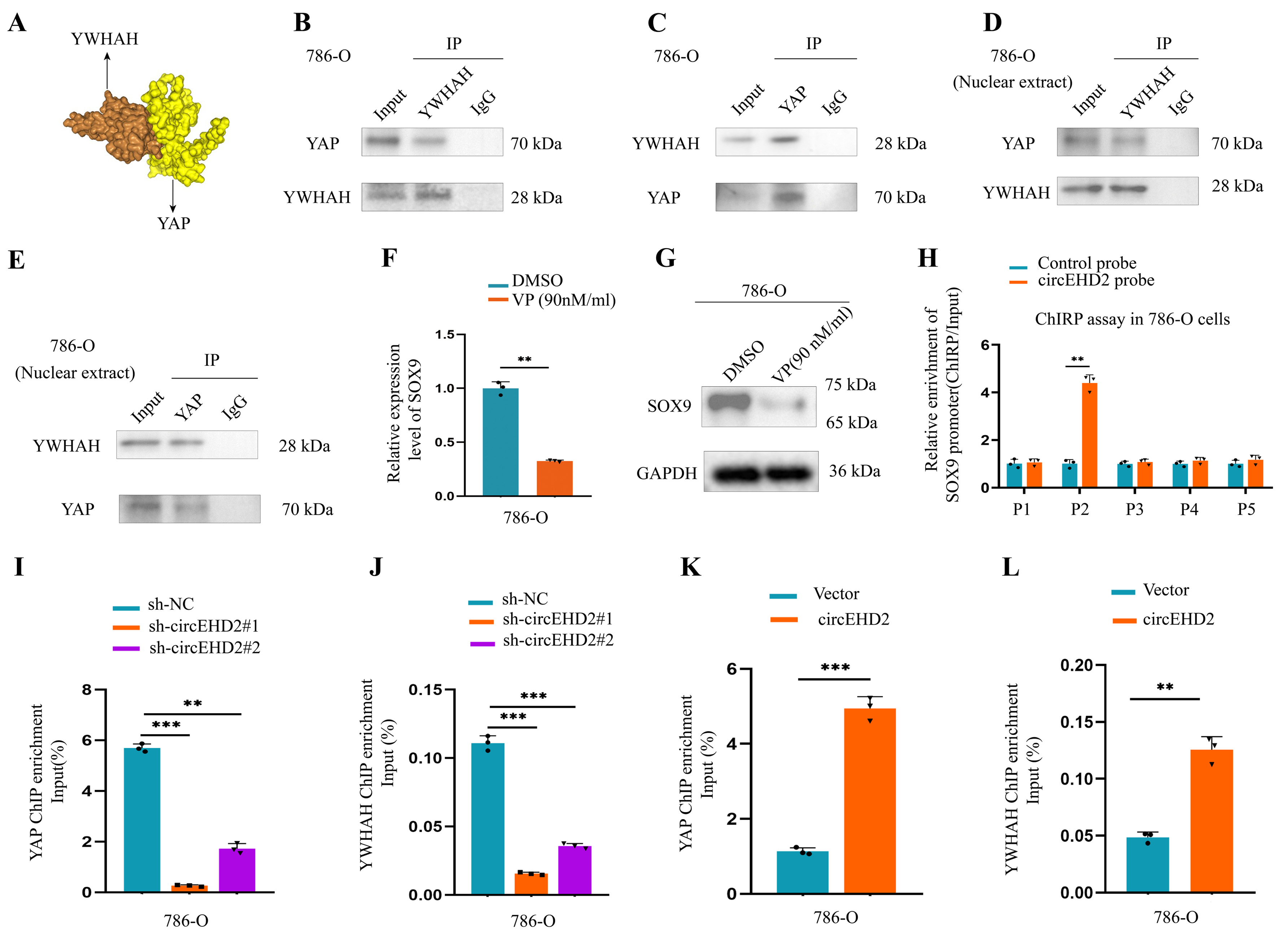


**Fig S8. circEHD2 promotes SOX9 expression by increasing YWHAH and YAP to the promoter of SOX9.** **A**, The three-dimensional structure showed that YWHAH could interact with YAP. **B** and **C**, Co-IP assay showed that YAP could be precipitated by anti-YWHAH antibody (**B**) and YWHAH could be precipitated by anti-YAP antibody **(C**) in 786-O cells. **D** and **E**, Co-IP assay confirmed that YWHAH could bind to YAP by anti-YWHAH antibody (**D**) and anti-YAP antibody (**E**) in the nuclear extracts of 786-O cells. F and G, qRT–PCR analysis (**F**) and western blot assay (**G)** showed the expression level of SOX9 in 786-O cells when cocultured with VP. **H**, ChIRP assay confirmed the enrichment of SOX9 promoter fragments in 786-O cells. **I–L**, ChIP-qPCR of YAP (**I** and **K**) and YWHAH (**J** and **L**) showed the enrichment of SOX9 promoter in 786-O cells with circEHD2 knockdown (**I** and **J**) or overexpression (K and L). Error bars represent the standard deviation (SD) of three independent experiments. **, *P*<0.01; ***, *P*<0.001.


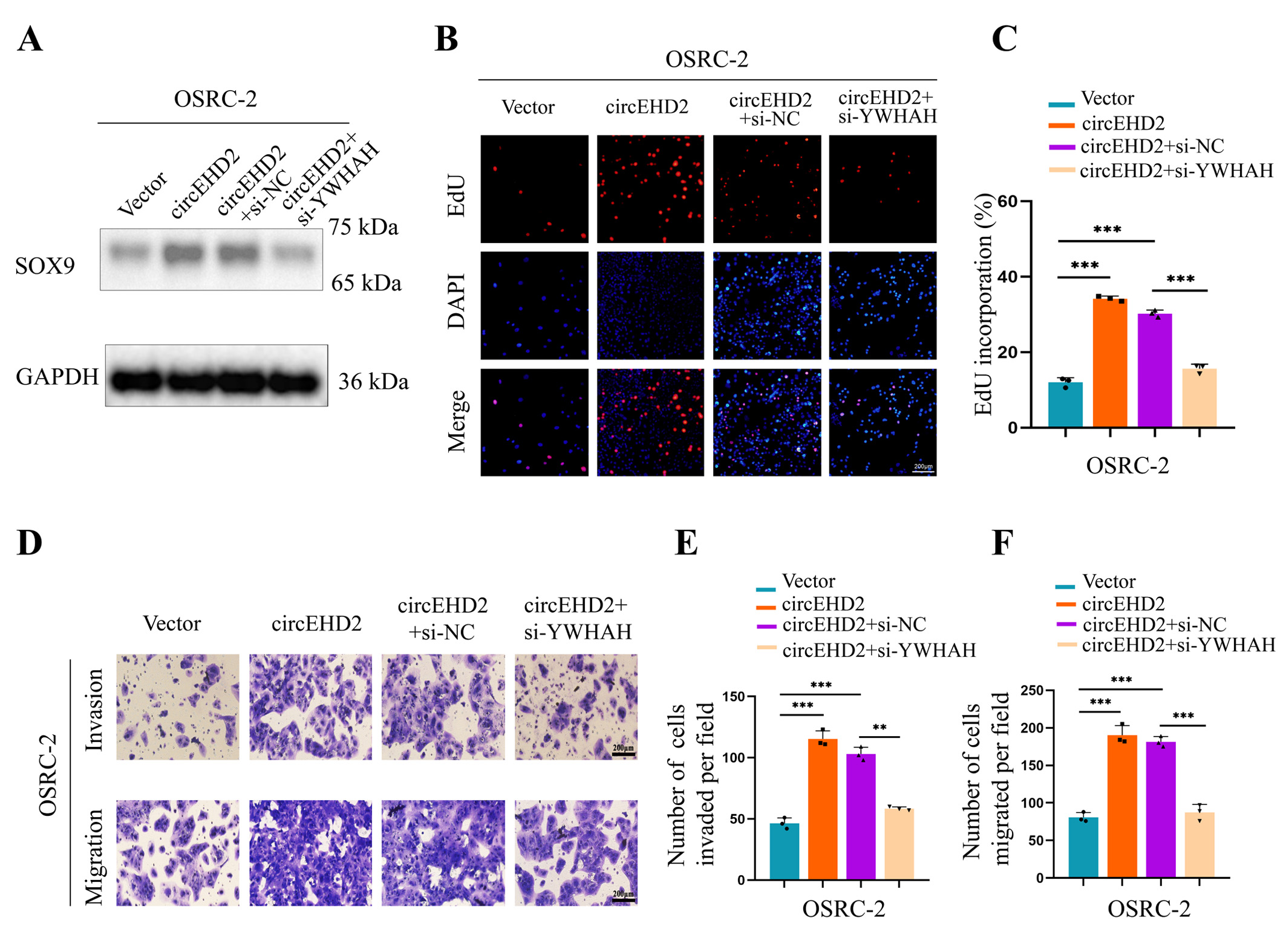


**Fig S9. YWHAH could partially reverse the tumor-promoting effects caused by the upregulation of circEHD2 in OSRC-2 cells.** **A**, Western blot analysis of the level of SOX9 in OSRC-2 cells after transfecting with circEHD2, followed by treatment with si-YWHAH. **B** and **C**, EdU assay showed that the proliferation ability was inhibited in OSRC-2 cells after transfecting with circEHD2, followed by treatment with si-YWHAH. Scale bars: 200μm. **D–F**, Transwell assays were conducted to measure the invasion and migration abilities of OSRC-2 cells after transfecting with circEHD2, followed by treatment with si-YWHAH. Scale bars: 200μm. Error bars represent the standard deviation (SD) of three independent experiments. **, *P*<0.01; ***, *P*<0.001.


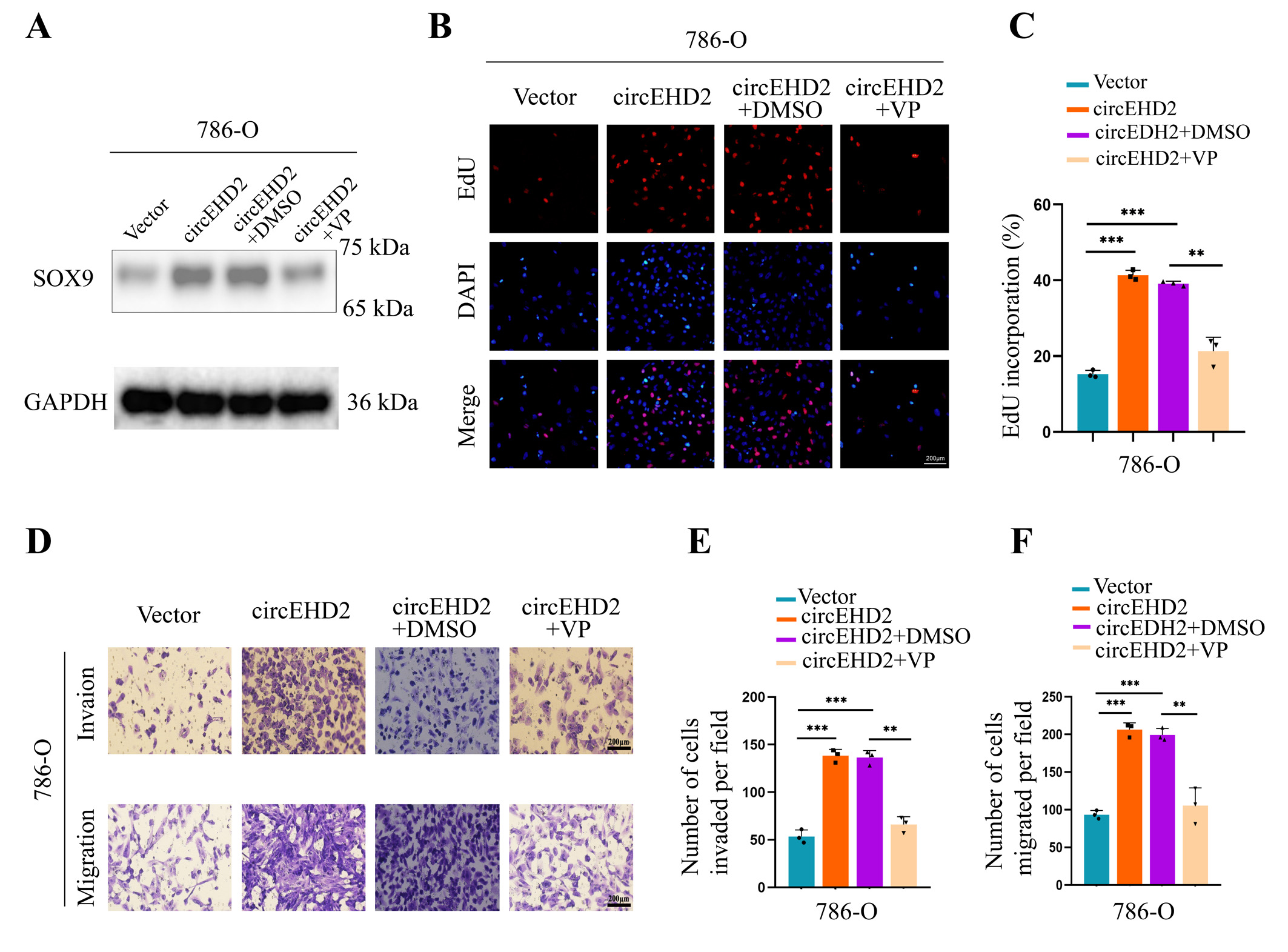


**Fig S10. Verteporfin (VP) could rescue the tumor-promoting effects caused by the upregulation of circEHD2 in 786- O cells. A,** Western blot analysis of the level of SOX9 in 786-O cells with circEHD2-overexpression after coculturing with VP. **B** and **C**, EdU assay showed that the proliferation ability was inhibited in 786-O cells with circEHD2-overexpression after treatment with VP. Scale bars: 200μm. **D–F**, Transwell assays were applied to measure the invasion and migration abilities of 786-O cells with circEHD2-overexpression after treating with VP. Scale bars: 200μm. Error bars represent the standard deviation (SD) of three independent experiments. **, *P*<0.01; ***, *P*<0.001.


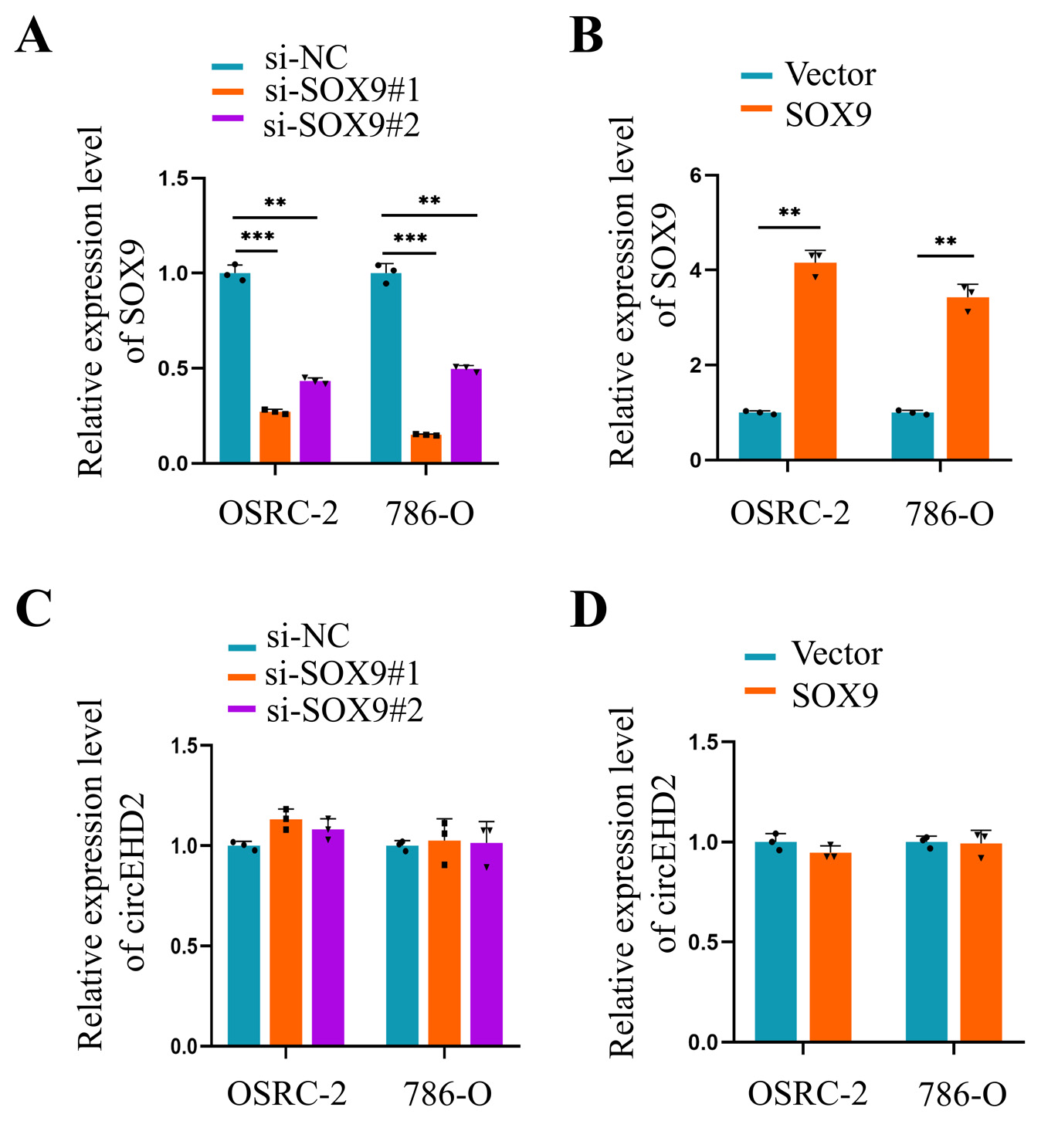


**Fig S11. The level of circEHD2 was not influenced by SOX9. A** and **B,** The knockdown efficiency (**A**) and overexpression (**B**) of SOX9 in OSRC-2 and 786-O cells measured by qRT-PCR. **C** and **D,** qRT-PCR assay revealed the level of circEHD2 was not affected by knockdown (**C**) or overexpress (**D**) of SOX9 in OSRC-2 and 786-O cells. Error bars represent the standard deviation (SD) of three independent experiments. **, *P*<0.01; ***, *P*<0.001.


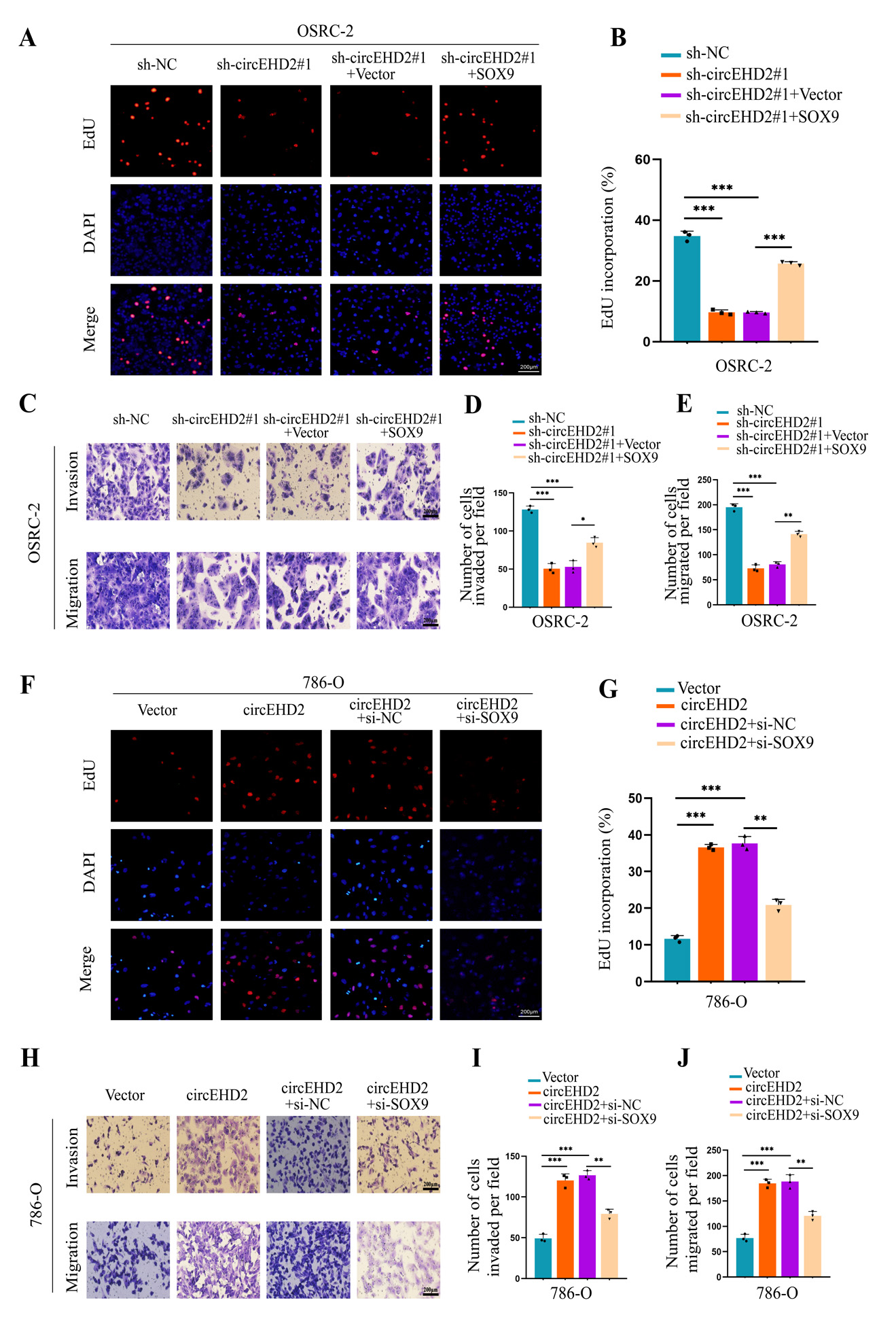


**Fig S12. SOX9 was crucial for the circEHD2-mediated progression of RCC**. **A** and **B**, EdU assay was performed to measure the proliferation ability of OSRC-2 cells with circEHD2 knockdown, followed by treated with vector and SOX9, respectively. Scale bars: 200μm. **C–E**, Transwell assays were performed to measure the invasion and migration abilities of OSRC-2 cells with circEHD2 knockdown, followed by treated with vector and SOX9, respectively. Scale bars: 200μm. **F** and **G**, EdU assay was performed to measure the proliferation ability of 786-O cells after transfecting with circEHD2, followed by treatment with si-NC and si-SOX9, respectively. Scale bars: 200μm. **H–J**, Transwell assays were performed to measure the invasion and migration abilities of 786-O cells after transfecting with circEHD2, followed by treatment with si-NC and si-SOX9, respectively. Scale bars: 200μm. Error bars represent the standard deviation (SD) of three independent experiments. *, *P*<0.05; **, *P*<0.01; ***, *P*<0.001.


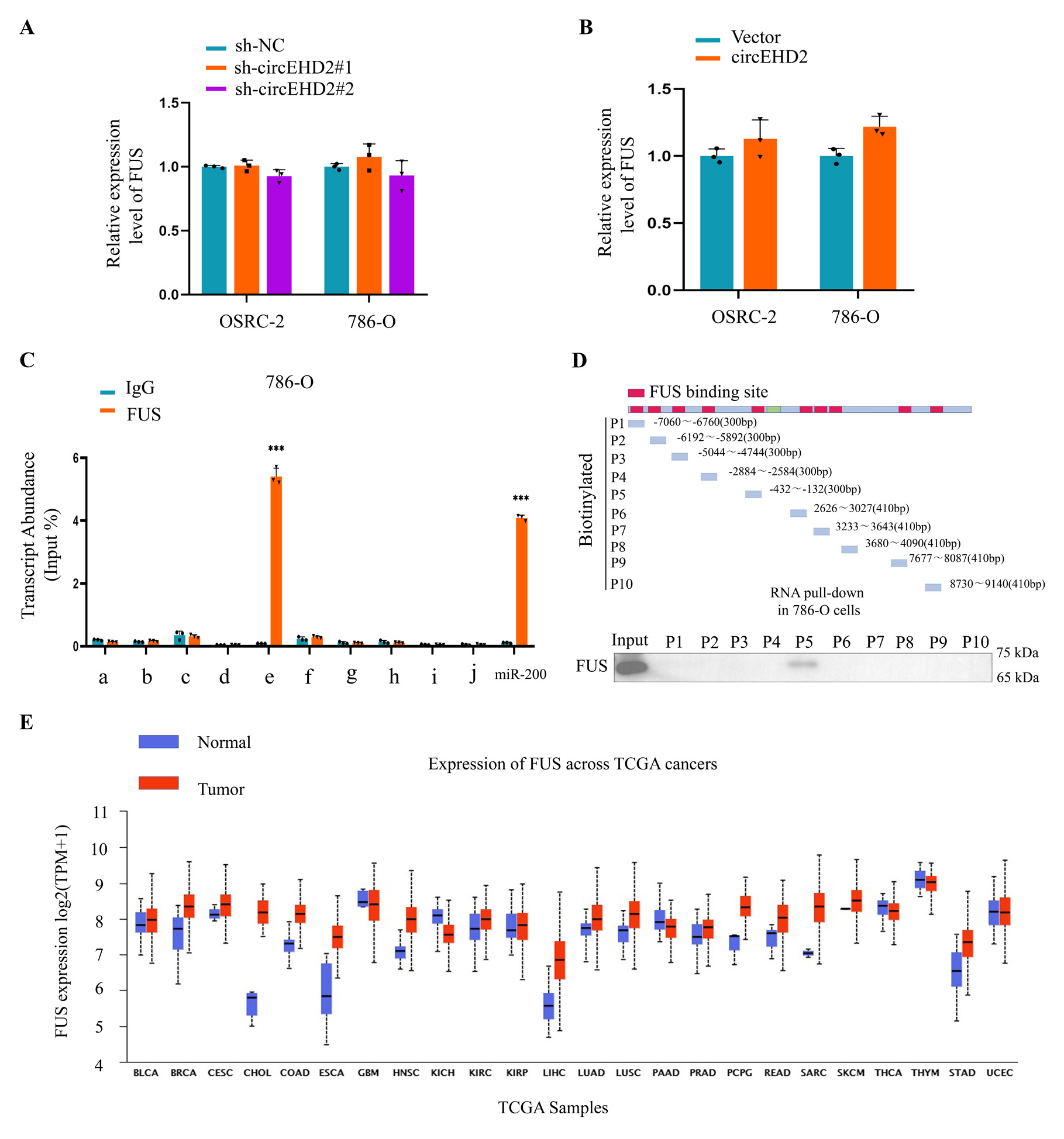


**Fig S13. FUS accelerates the cyclization of circEHD2. A** and **B,** qRT-PCR assay showed that the level of FUS was not influenced by the knockdown (**A**) or overexpression (B) of circEHD2 in OSRC-2 and 786-O cells. **C**, The RIP assay was used to validate the binding sites of FUS on circEHD2 pre-mRNA in 786-O cells. miR-200 was used as the positive control. **D**, The RNA pull-down assay was used to confirm the interaction between FUS and the putative 10 fragments of circEHD2 pre-mRNA (P1-P10) in 786-O cells. **E**, FUS was upregulated in most cancers from the TCGA database. Error bars, standard deviation (SD) of three independent experiments. ***, *P*<0.001.


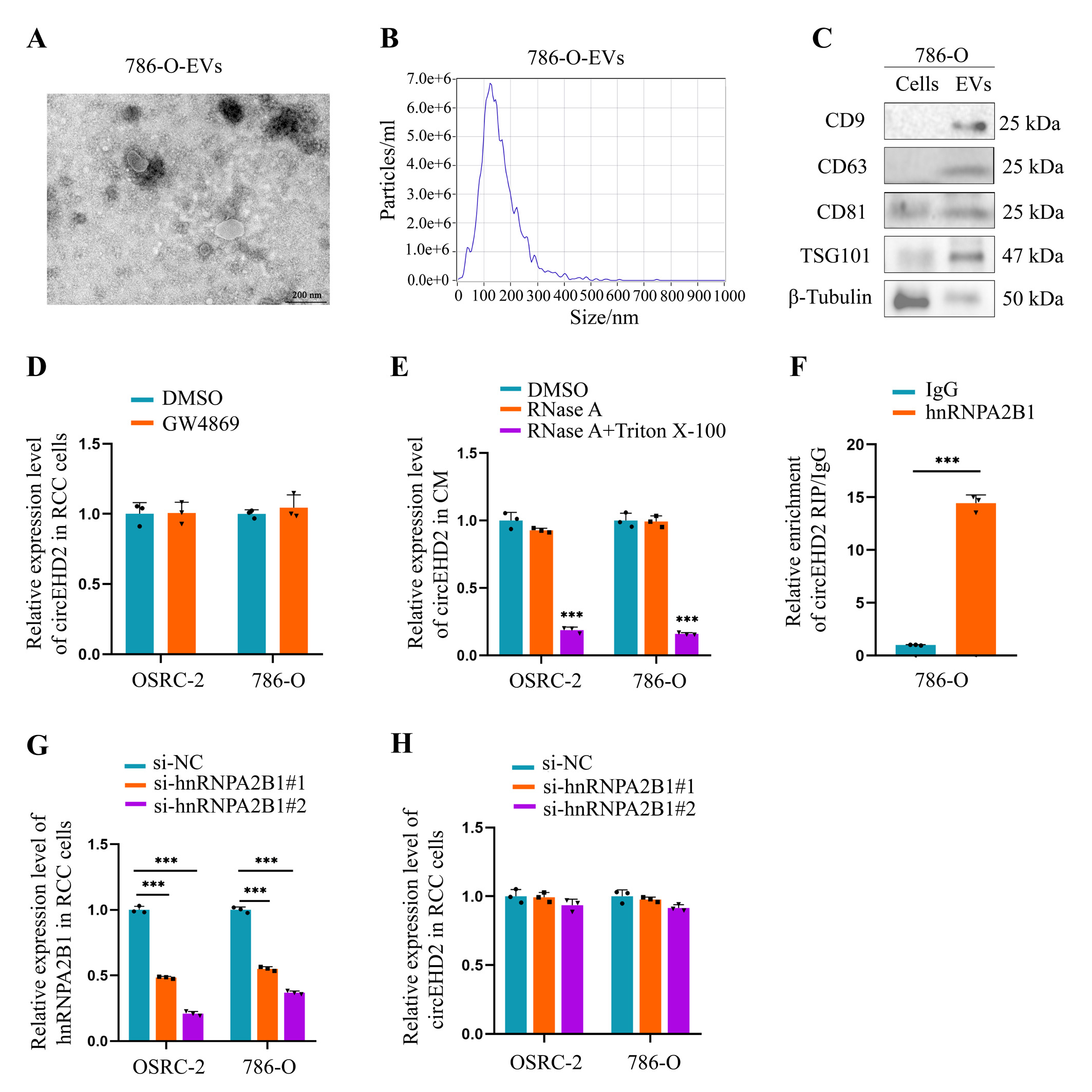


**Fig S14. hnRNPA2B1 was crucial for the packaging of circEHD2 into EVs. A** and **B,** TEM and NanoSight were used to identify the character of EVs purified from 786-O cells. Scale bars: 200 nm. **C**, Western blot analysis of EVs markers from 786-O EVs or cell lysates. **D**, qRT-PCR analysis of circEHD2 expression in RCC cells after treatment with GW4869. **E**, qRT-PCR analysis of the level of circEHD2 in the culture medium of RCC cells treated with RNase A (2 mg/mL) or in combination with Triton X-100 (0.1%) for 30 minutes. **F**, RIP assay in 786-O cells confirmed that circEHD2 could be enriched by hnRNPA2B1. **G**, The knockdown efficiency of hnRNPA2B1 in RCC cells measured by qRT-PCR. **H**, qRT-PCR analysis of circEHD2 expression in RCC cells after knockdown of hnRNPA2B1. Error bars, standard deviation (SD) of three independent experiments. ***, *P*<0.001.


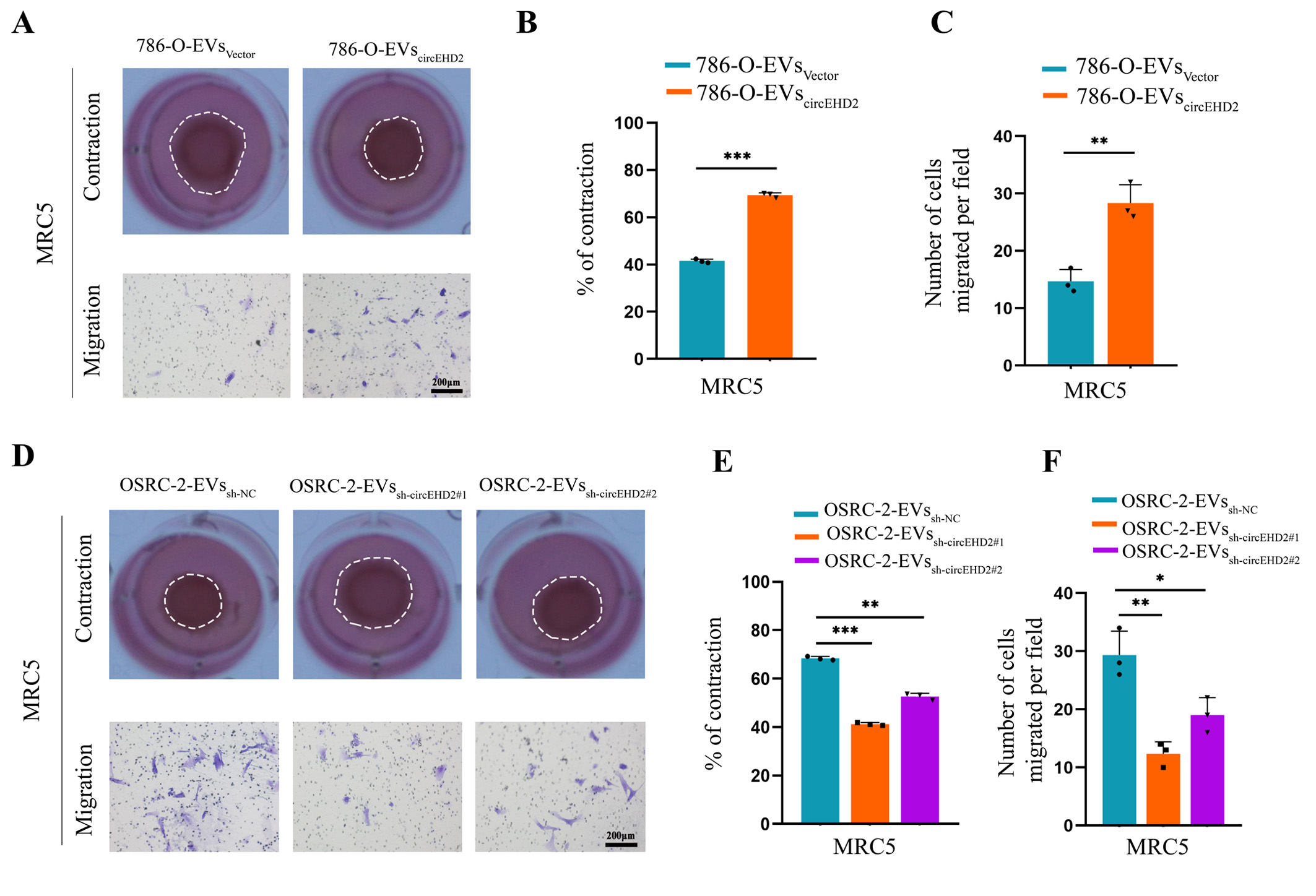


**Fig S15. EVs-circEHD2 promotes collagen contraction and migration of MRC5 cells in vitro.** **A-C**, Representative images of MRC5 cells cultured with 786-O-EVs_Vector_ and 786-O-EVs_circEHD2_. Scale bars: 200μm. (**B**) The collagen contraction and (**C**) the number of migrated cells. **D-F**, Representative images of MRC5 cells cultured with OSRC-2-EVs_sh-NC_, OSRC-2-EVs_sh-circEHD2#1_ and OSRC-2-EVs_sh-circEHD2#2_. Scale bars: 200μm. (**E**) The collagen contraction and (**F**) the number of migrated cells. Error bars, standard deviation (SD) of three independent experiments. *, *P*<0.05; **, *P*<0.01; ***, *P*<0.001.


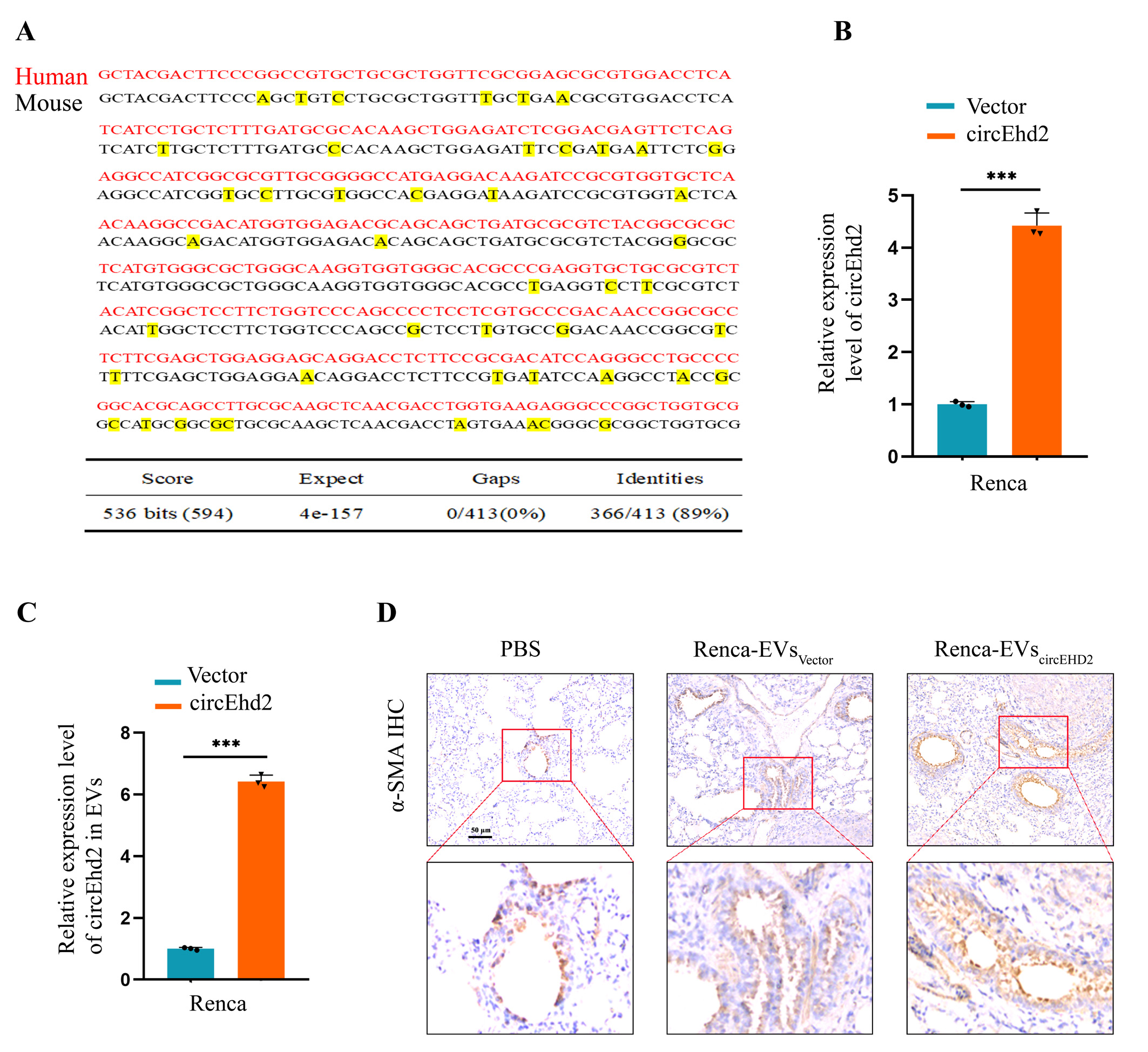


**Fig S16. EVs-circEHD2 promotes metastasis of RCC.** **A**, circEHD2 homology analysis showed high homology (89%) in human and mouse circEHD2 through the Basic Local Alignment Search Tool (BLAST). **B**, qRT-PCR analysis of the overexpression efficiency of circEhd2 in Renca cells. **C**, qRT-PCR analysis of the expression level of EVs-circEhd2 in Renca cells with circEhd2 overexpression. **D**, Images of IHC staining by anti-α-SMA antibody in metastasis lung after treatment with PBS, Renca-EVs_Vector_, and Renca-EVs_circEHD2_. Scale bars: 50μm. Error bars, standard deviation (SD) of three independent experiments. ***, *P*<0.001.


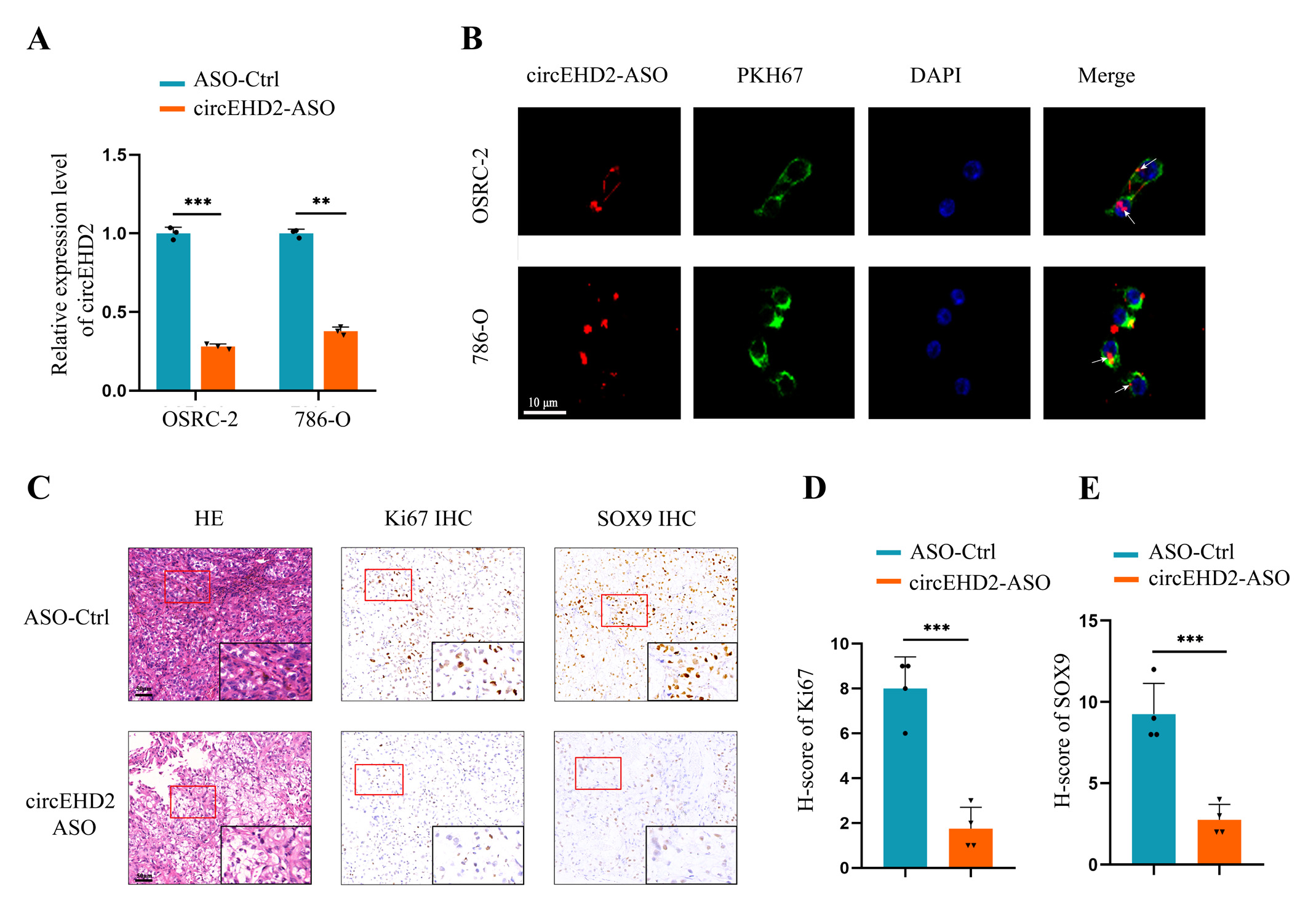


**Fig S17. circEHD2-ASO could be internalized by RCC cells. A**, qRT-PCR analysis of the inhibitory efficiency of circEHD2-ASO in RCC cells. **B**, Representative images of OSRC-2 and 786-O cells after incubation with circEHD2-ASO labeled with CY3, PKH67 was used to label the cytomembrane. Scale bars: 10μm. **C**, Images of HE and IHC staining by anti-Ki67 antibody, and anti-SOX9 antibody in orthotopic tumors after treating with circEHD2-ASO (n = 4/group). Scale bars: 50μm. **D** and **E**, H-score of Ki67 (**D**) and SOX9 (**E**) in orthotopic tumors after treating with circEHD2-ASO (n = 4/group). Error bars represent the standard deviation (SD) of three independent experiment*s.* **, *P*<0.01; ***, *P*<0.001.Table S1. The correlation between circEHD2 expression and clinicopathologic characteristics in 80 ccRCC patients.

| Characteristics | Patients (80) | circEHD2 expression | | *P*-value |
| --- | --- | --- | --- | --- |
|  |  | High group | Low group |  |
| Age (years) |  |  |  | 0.3709 |
| >58 | 41 | 18 | 23 |  |
| ≤58 | 39 | 22 | 17 |  |
| Gender |  |  |  | 1 |
| Male | 56 | 28 | 28 |  |
| Female | 24 | 12 | 12 |  |
| Tumor stage |  |  |  | 0.0121 |
| Ⅰ/Ⅱ | 48 | 18 | 30 |  |
| Ⅲ/Ⅳ | 32 | 22 | 10 |  |
| Tumor grade |  |  |  | 0.0005 |
| Ⅰ/Ⅱ | 50 | 17 | 33 |  |
| Ⅲ/Ⅳ | 30 | 23 | 7 |  |
| Metastasis status |  |  |  | 0.1610 |
| Metastasis | 16 | 11 | 5 |  |
| Non-metastasis | 64 | 29 | 35 |  |

Table S2. The sh-RNAs, si-RNAs and ASO used in this study

| sh-RNAs or si-RNAs | Sequence(5’-3’) |
| --- | --- |
| sh-NC | TTCTCCGAACGTGTCACGT |
| sh-circEHD2#1 | GGCTGGTGCGAGCTACGACTT |
| sh-circEHD2#2 | GTGCGAGCTACGACTTCCCGG |
| si-YWHAH#1 | GCAATGATTTCCAGTATGA |
| si-YWHAH#2 | CCAGAATGCACCTGAGCAA |
| si-SOX9#1 | CGCTCACAGTACGACTACA |
| si-SOX9#2 | CCACCTTCACCTACATGAA |
| si-FUS#1 | CAAGCAGATTGGTATTATT |
| si-FUS#2 | CACGGACACTTCAGGCTAT |
| si-hnRNPA2B1#1 | GCTACGGAGGTGGTTATGA |
| si-hnRNPA2B1#2 | GGAGAGTAGTTGAGCCAAA |
| circEHD2-ASO | GCTGGTGCGAGCTACGACTT |

Table S3. Primes used in qRT-PCR and PCR analysis in this study

| Primers | Sequence(5’-3’) |
| --- | --- |
| GAPDH | F: CAGGAGGCATTGCTGATGAT |
|  | R: GAAGGCTGGGGCTCATTT |
| Gapdh (mouse) | F: GGTTGTCTCCTGCGACTTCA |
|  | R: TGGTCCAGGGTTTCTTACTCC |
| U6 | RT:GTCGTATCCAGTGCAGGGTCCGAGGTATTCGCACTGGATACGACAAAATA |
|  | F: CTCGCTTCGGCAGCACA |
|  | R: AACGCTTCACGAATTTGCGT |
| circEHD2 | F: CTGGTGCGAGCTACGACTTC |
|  | R: TCGTCCGAGATCTCCAGCTT |
| circEhd2 (mouse) | F: GTCCTTCGCGTCTACATTGG |
|  | R: AGCTGCTGTGTCTCCACCAT |
| hsa_circ_0006528 | F: GGAGTCACTGCCTTACGTGGA |
|  | R: ACTGGGAAGACAGGAGGCAG |
| hsa_circ_0072954 | F: TGCCAAGTGTGTAGAACAGGAG |
|  | R: TGTCATTGACCTGGAGTATGCC |
| hsa_circ_0072732 | F: CCTCGTGACTGGAGAGAACA |
|  | R: TGAGCTTTGAACTTTTCCTCTG |
| has_circ_0002484 | F: GAGTGATCAGCAGACAGTTCCAGG |
|  | R: GCTGTGGGAAGAGGAGCTATGAG |
| EHD2 mRNA | F: CTCTGCCTACCACCATTCCA |
|  | R: GGGCTGTAGCACCAAGAACC |
| circEHD2 (divergent) | F: CTGGTGCGAGCTACGACTTC |
|  | R: TCGTCCGAGATCTCCAGCTT |
| circEHD2 (convergent) | F: AGTTCTCAGAGGCCATCGGC |
|  | R: ACCAGAAGGAGCCGATGTAGAC |
| GAPDH (convergent) | F: CAGGAGGCATTGCTGATGAT |
|  | R: GAAGGCTGGGGCTCATTT |
| Rolling circle amplification (circEHD2) | F: AGTTCTCAGAGGCCATCGGC |
|  | R: ACCAGAAGGAGCCGATGTAGAC |
| YWHAH | F: TGGGCATTGCTGGACTGAT |
|  | R: CTTTCGCCTAGATTTCCCATAGAT |
| YAP | F: AGGAGACACATGCACCGGA |
|  | R: CAGCAGCAATGGACAAGGAA |
| SOX9 | F: AGGAGAGCGAGGAGGACAAGTTC |
|  | R: CTGCCCGTTCTTCACCGACTTC |
| FUS | F: AAGTGTCCTAATCCCACCTGTGA |
|  | R: GGGCCTTACACTGGTTGCATT |
| hnRNPA2B1 | F: CAACCTTCTAACTACGGTCCAA |
|  | R: CAGTATCGGCTCCTCCCAC |
| CFTR | F: TTTCCTCCAAACCTCACAGCAACTC |
|  | R: CTCGGCTCACAGATCGCATCAAG |
| PDE4B | F: AATCTCACCAAGAAGCAGCGTCAG |
|  | R: TCCTCCATGATGCGGTCTGTCC |
| RYR2 | F: TGACAACTCCTTCCTCTACCTAGCC |
|  | R: CCTCCAGCACGAACTCCAACATAC |
| GRIN2A | F: AGACCAGATGCTTCAGGAGACAGG |
|  | R: TGGGAGTGGAGGAAAGGGTTATCG |
| MYL9 | F: TGATGGCTTCATTGACAAGGAGGAC |
|  | R: ATGTTTGAGGATGCGGGTGAACTC |
| FXYD2 | F: GGGGCGGTAAGAAACATAGGC |
|  | R: CAACTTGGAACAGGGAGTGGG |
| NYP1R | F: TGCTTGTTGCCATCATGTGTCTCC |
|  | R: AAGCCACAGCAAGGACCCAAATC |
| CACNA1D | F: TGTACGATGGCATCATGGCTTACG |
|  | R: CTCCTCCTCTTCCTCTTCCTCTTCC |
| HCAR3 | F: GCCGTTCGTGATGGACTACTATGTG |
|  | R: GCAACTTCTTCTTCAGGAGGTGGAC |
| HCAR2 | F: TCGTCTTTGTCATCTGCTTCCTTCC |
|  | R: TGCTGCGGTTATTATCTGGCTCAC |
| PIK3R1 | F: CTGTTGCGAGGGAAGCGAGATG |
|  | R: AAGGGAGGTGTGTTGGTAATGTAGC |
| SOX9 (P1)  ChIRP | F: TGCTGTGCGTTTATTTGGGATTCTG |
|  | R: GACCTGCATGTAGTTGGGAGTTCTG |
| SOX9 (P2) ChIRP | F: GCAAGCATGTGTCATCCATATTTC |
|  | R: CCTCAAACTCTCTAGCCACAGCA |
| SOX9 (P3) ChIRP | F: GGGCGTTGAGTCACCAAAACATTTG |
|  | R: AAGTTTGTCGTACTCTCGGAATGCC |
| SOX9 (P4) ChIRP | F: GGGACTGCTGTGCTGTGATTGG |
|  | R: GGCGAATTGGAGAGGAGGAGGAG |
| SOX9 (P5) ChIRP | F: ATGAAGATGACCGACGAGCA |
|  | R: TCGCTCTCCTTCTTCAGATCG |
| SOX9 (ChIP) | F: TCCGCACTTACCCAACCTG |
|  | R: GGCATTGGTGGTGTCTCTCAT |
| circEHD2 pre-mRNA (a) | F: GGGTCTGGACTCCTGGATCTGAG |
|  | R: CCAGAGGACACAGGCCCAAGTC |
| circEHD2 pre-mRNA(b) | F: GCAGCTGCAGAAACGATTGG |
|  | R: CTCTATCCTCAAGGCCAGGGA |
| circEHD2 pre-mRNA(c) | F: TTTTGTGGGACTGTGTGTGACTACC |
|  | R: CACACGGCATAGACACAGAGACC |
| circEHD2 pre-mRNA(d) | F: TCTAGCTGGAGGGCATGAAGTCAG |
|  | R: TCCTTACATCTCTCAGCCCACTGG |
| circEHD2 pre-mRNA(e) | F: AATTAGCCAGGTGTGGTGATGCG |
|  | R: GCTCTCCAGTGCCCAGAATTTCC |
| circEHD2 pre-mRNA(f) | F: ACGATGGTTCCAGTCCTGTCCTG |
|  | R: ATTTCCCCTCAGATGCCCTCTCC |
| circEHD2 pre-mRNA(g) | F: AGGCTTGGAGGTTCCTGGATCAG |
|  | R: CCCACTCTCGCTGCTCCTTAAAC |
| circEHD2 pre-mRNA(h) | F: CTGCCTCCTCCTGTCTCTCCATC |
|  | R: AGGTGCCAGGAAGAGATAAGGTGAG |
| circEHD2 pre-mRNA(i) | F: ACTTCGTGAATTGCTGCTGTAGGG |
|  | R: CAGTGGCACAATCTCGGCTCAG |
| circEHD2 pre-mRNA(j) | F: GGTGGAGGTTGCAGTGAGACAAG |
|  | R: CCCTAAGTGGAATTTGAGCCTAGCC |
| miR-200 | RT:GTCGTATCCAGTGCAGGGTCCGAGGTATTCGCACTGGATACGACTCCATC |
|  | F: CGCGTAATACTGCCGGGTAAT |
|  | R: AGTGCAGGGTCCGAGGTATT |

Table S4. Probes of FISH, RNA pull-down, ChIRP and ISH in this study

| Probes | Sequence(5’-3’) |
| --- | --- |
| circEHD2 FISH probe | AGCACGGCCGGGAAGTCGTAGCTCGCACCA |
|  | 5’-CY3 labeled 3’-CY3 labeled |
| Biotin labeled control probe  (RNA pull-down) | GTAGATCAGTAGGTTGATCGTAGATAGAAG |
| Biotin labeled circEHD2 probe  (RNA pull-down) | AGCACGGCCGGGAAGTCGTAGCTCGCACCA |
| circEHD2 ChIRP control probe | GTAGATCAGTAGGTTGATCGTAGATAGAAG |
| circEHD2 ChIRP probe | AGCACGGCCGGGAAGTCGTAGCTCGCACCA |
| circEHD2 ISH probe | AGCACGGCCGGGAAGTCGTAGCTCGCACCA  5’-DIG labeled and 3’-DIG labeled |
| Scramble ISH probe (negative control) | GTAGATCAGTAGGTTGATCGTAGATAGAAG 5’-DIG labeled and 3’-DIG labeled |
| U6 ISH probe | CACGAATTTGCGTGTCATCCTT  5’-DIG labeled and 3’-DIG labeled |

Abbreviation: DIG, Digoxigenin.

Table S5. The co-upregulated circRNAs from GSE100186 and GSE108735.

| CircRNA ID | Chromesome | Location |
| --- | --- | --- |
| hsa_circ_0003146 | 19 | chr19:48229068-48229481 |
| hsa_circ_0006528 | 5 | chr5:145197456-145205763 |
| hsa_circ_0072954 | 5 | chr5:72285253-72286691 |
| hsa_circ_0072734 | 5 | chr5:65349233-65350779 |
| hsa_circ_0002484 | 11 | chr11:130130750-130131824 |
